# Effect of Maternal and Newborn Care Service Package on Perinatal and Newborn Mortality: A Cluster Randomized Clinical Trial

**DOI:** 10.1001/jamanetworkopen.2023.56609

**Published:** 2024-02-19

**Authors:** Shabina Ariff, Uswa Jiwani, Arjumand Rizvi, Sajid Muhammad, Amjad Hussain, Imran Ahmed, Masawar Hussain, Muhammad Usman, Junaid Iqbal, Zahid Memon, Sajid Bashir Soofi, Zulfiqar A. Bhutta

**Affiliations:** 1 Department of Paediatrics and Child Health, Aga Khan University, Karachi, Pakistan; 2 Center of Excellence in Women and Child Health, Aga Khan University, Karachi, Pakistan; 3 Institute of Global Health and Development, Aga Khan University, Karachi, Pakistan

## Abstract

**Importance:**

In resource-constrained settings where the neonatal mortality rate (NMR) is high due to preventable causes and health systems are underused, community-based interventions can increase newborn survival by improving health care practices.

**Objectives:**

To develop and evaluate the effectiveness of a community-based maternal and newborn care services package to reduce perinatal and neonatal mortality in rural Pakistan.

**Design, Setting, and Participants:**

This cluster randomized clinical trial was conducted between November 1, 2012, and December 31, 2013, in district Rahim Yar Khan in the province of Punjab. A cluster was defined as an administrative union council. Any consenting pregnant resident of the study area, regardless of gestational age, was enrolled. An ongoing pregnancy surveillance system identified 12 529 and 12 333 pregnancies in the intervention and control clusters, respectively; 9410 pregnancies were excluded from analysis due to continuation of pregnancy at the end of the study, loss to follow-up, or miscarriage. Participants were followed up until the 40th postpartum day. Statistical analysis was performed from January to May 2014.

**Intervention:**

A maternal and newborn health pack, training for community- and facility-based health care professionals, and community mobilization through counseling and education sessions.

**Main Outcomes and Measures:**

The primary outcome was perinatal mortality, defined as stillbirths per 1000 births and neonatal death within 7 days per 1000 live births. The secondary outcome was neonatal mortality, defined as death within 28 days of life per 1000 live births. Systematic random sampling was used to allocate 10 clusters each to intervention and control groups. Analysis was conducted on a modified intention-to-treat basis.

**Results:**

For the control group vs the intervention group, the total number of households was 33 188 vs 34 315, the median number of households per cluster was 3092 (IQR, 3018-3467) vs 3469 (IQR, 3019-4075), the total population was 229 155 vs 234 674, the mean (SD) number of residents per household was 6.9 (9.5) vs 6.8 (9.6), the number of males per 100 females (ie, the sex ratio) was 104.2 vs 103.7, and the mean (SD) number of children younger than 5 years per household was 1.0 (4.2) vs 1.0 (4.3). Altogether, 7598 births from conrol clusters and 8017 births from intervention clusters were analyzed. There was no significant difference in perinatal mortality between the intervention and control clusters (rate ratio, 0.86; 95% CI, 0.69-1.08; *P* = .19). The NMR was lower among the intervention than the control clusters (39.2/1000 live births vs 52.2/1000 live births; rate ratio, 0.75; 95% CI, 0.58-0.95; *P* = .02). The frequencies of antenatal visits and facility births were similar between the 2 groups. However, clean delivery practices were higher among intervention clusters than control clusters (63.2% [2284 of 3616] vs 13.2% [455 of 3458]; *P* < .001). Chlorhexidine use was also more common among intervention clusters than control clusters (55.9% [4271 of 7642] vs 0.3% [19 of 7203]; *P* < .001).

**Conclusions and Relevance:**

This pragmatic cluster randomized clinical trial demonstrated a reduction in NMR that occurred in the background of improved household intrapartum and newborn care practices. However, the effect of the intervention on antenatal visits, facility births, and perinatal mortality rates was inconclusive, highlighting areas requiring further research. Nevertheless, the improvement in NMR underscores the effectiveness of community-based programs in low-resource settings.

**Trial Registration:**

ClinicalTrials.gov Identifier: NCT01751945

## Key Points

**Question:**

Can packaged community-based interventions delivered through existing health care systems improve neonatal survival in resource-constrained regions?

**Findings:**

This pragmatic, cluster randomized clinical trial in Pakistan included 15 615 births and an intervention package consisting of community mobilization through health education, provision of clean delivery kits to pregnant women, and training community- and facility-based health care professionals. Although the intervention showed no reduction in perinatal mortality, there was a statistically significant 25% reduction in neonatal mortality.

**Meaning:**

This trial demonstrates a high level of acceptance for improved household practices around delivery and neonatal care by community members and supports the use and scale-up of community-based approaches to improve neonatal mortality in Pakistan.

## Teaser

This pragmatic cluster randomized clinical trial evaluates the effectiveness of a community-based maternal and newborn care services package to reduce perinatal and neonatal mortality in rural Pakistan.

## Introduction

In 2020, the global neonatal mortality rate (NMR) stood at 17 deaths per 1000 live births, resulting in 2.4 million fatalities.^1^ Despite extensive efforts to improve newborn survival, neonatal mortality remains a significant public health concern, contributing to nearly half of all deaths of children younger than the age of 5 years in 2020. Projections indicate that between 2020 and 2030, approximately 24 million newborn deaths are expected, with most concentrated in sub-Saharan Africa and South Asia.^2^

At 40 deaths per 1000 live births, Pakistan has one of the highest NMRs in the world.^3^ Most newborn deaths are preventable through proper care during delivery and the postpartum period; however, barriers within health systems significantly impede the uptake of these essential services. These barriers include inadequate health care facilities, shortage of health care professionals, and poor care-seeking practices due to a lack of awareness, perceived suboptimal quality of health services, and high out-of-pocket costs.^4,5,6,7,8^ In Pakistan, 41% of women deliver at home in rural areas, and 37% of newborns are born without skilled birth attendants.^9^ Fewer than half the mothers in rural areas (49%) receive a postnatal visit by a skilled clinician, and only 52% of newborns are examined by a qualified clinician within 2 days of birth.^9^ Furthermore, nearly three-fourths (74.9%) of women in rural areas face at least 1 challenge in accessing health facilities, with reluctance to travel alone and distance to health facilities being the most commonly reported barriers.^9^

In areas of poor accessibility, equipping outreach professionals, such as community health workers, to provide necessary services can reduce neonatal morbidity and mortality.^10,11,12,13^ At the same time, interventions encouraging mothers to seek care by educating them and their families on safe motherhood and facility births,^14,15^ providing incentives, and improving referral systems can increase the uptake of antenatal and early postnatal care.^16,17^ This study aimed to determine if an integrated health services package that focused on demand creation within the community and delivery of lifesaving interventions through existing community-based health care professionals could improve neonatal mortality in rural Pakistan.

## Methods

### Study Design and Setting

A community-based, pragmatic, parallel, 2-group, cluster randomized clinical trial was conducted in Tehsil Rahim Yar Khan (RYK), a subdivision of district RYK in Punjab province, Pakistan. Baseline surveys and health facility assessments were conducted between April 1 and October 31, 2012, while enrollment and intervention delivery occurred between November 1, 2012, and December 31, 2013. The predominantly rural Tehsil RYK has a population of 1.4 million people and is administratively divided into 40 union councils (UCs), each with a population of approximately 15 000 to 20 000 people and at least 1 basic health unit that provides primary care services to the UC. Each UC is also served by lady health workers (LHWs), community health workers employed by the government, who provide door-to-door primary, preventative, and curative care to men, women, and children, with a focus on reproductive, maternal, and child health, and act as liaisons between the community and health facilities. District RYK has a high NMR of 39 deaths per 1000 live births.^18^ The protocol for this study has been previously published (trial protocol in Supplement 1).^19^ The study was approved by the ethics review committee of Aga Khan University and the national bioethics committee of Pakistan. Verbal consent was obtained from respondents and heads of households before data collection. This study is reported according to the Consolidated Standards of Reporting Trials (CONSORT) reporting guideline.

### Participants, Sample Size, Randomization, and Masking

Pregnant women, regardless of gestational age, residing in the study area, identified through an ongoing pregnancy surveillance system instituted in the study area and LHW records, were eligible to participate. A cluster was defined as an administrative UC. Assuming each cluster has a population of 15 000 to 20 000 people, a crude annual birth rate of 20 births per 1000 people, and an estimated perinatal mortality rate of 60 deaths per 1000 births, a coefficient of variation (κ) between clusters of 0.125 and an intracluster correlation coefficient of 0.05 were used. A total of 20 clusters were required to detect a difference of 20% in mortality rates between the intervention and control groups with 90% power.

There were 10 clusters each in the intervention and control groups (eFigure 1 in Supplement 2). Blocked randomization of clusters was conducted after stratifying the clusters. As a result, 139 random allocations were identified that resulted in similar populations in the 2 groups (difference, <20 000), number of live births (difference, <1000), NMRs (difference, <10 deaths per 1000 live births), and proportions of women delivering in a hospital (difference, <5%). One scheme was selected from this list of balanced allocations using a computer-generated random number to allocate clusters to their respective groups. Due to the nature of the study (provision of intervention vs no intervention), blinding was not possible. However, to reduce bias in the study, independent data collection teams masked to cluster allocation were established to collect data on the outcomes.

### Procedures

An evidence-based intervention package was developed comprising a maternal and newborn health pack, training for community health workers (including LHWs and community midwives), training for facility-based health care professionals, and community mobilization through health education. The evidence taken into consideration for each component of the package is presented in eTable 2 in Supplement 2.

To assess the preintervention health indicators of the study area, tailor the health education component for the community, and develop training modules for clinicians, a comprehensive baseline survey was conducted. Sociodemographic characteristics, maternal mortality rates and NMRs within the past year, and health care practices during prior pregnancies, including the number of facility-based births, clean deliveries, and deliveries performed by skilled attendants, were obtained. Clean delivery was defined as clean hands, perineum, surface, cord cutting, tying, and avoiding introduction of unclean materials into the vagina.^20^ Nested within the baseline survey, a study on knowledge, attitudes, and practices explored the community’s understanding, practices, and health-seeking behaviors related to maternal and newborn health and informed the development of the health education component. A health facility assessment was conducted to collect information on staffing, level of care provided, and availability of laboratory, blood bank, transportation facilities, labor and delivery room, postnatal wards, and nursery. These data informed the development of clinical training modules for the health care professionals tailored to the available services.

#### Intervention Package

##### Maternal and Newborn Health Pack

The health pack contained a 4% chlorhexidine solution, sunflower oil emollient, and a clean delivery kit (CDK) with a soap bar, disposable gloves, a clean plastic sheet, a single-use razor blade, a cord clamp, and sterile thread. Pictorial brochures in the local language provided instructions on CDK use, key health messages on birth preparedness, maternal and newborn danger signs, acute obstetric and newborn emergencies, and immediate newborn care.

##### Enhanced Training

A detailed description of the training provided to each group of health care workers is presented in eTable 1 in Supplement 2. In brief, LHWs and community midwives in the intervention clusters received refresher training on recognizing complications and danger signs among mothers and neoates, providing prompt referrals, and the importance and use of CDKs. Clinicians in primary and secondary health care facilities received refresher training on basic and comprehensive emergency obstetric and neonatal care.^21^

##### Community Mobilization

To foster maternal and newborn health awareness within the community, LHWs facilitated informational sessions at the household and community levels for pregnant women and their families in the intervention clusters. Participants learned about antenatal nutrition, vaccination, pregnancy complications, maternal and neonatal danger signs, and essential and immediate newborn care. Pregnant women were encouraged to seek antenatal and postnatal care and to deliver in a health care facility or with a skilled birth attendant in case of a home birth. These messages were also reiterated via text messages sent to pregnant women and their families throughout the pregnancy and postpartum period.

An emergency fund was also established with the help of LHWs using local resources and contributions from pregnant women’s families. This fund was used to transport women at high risk or with other complications to health care facilities.

#### Study Teams

Twenty independent data collection teams were formed, 1 for each cluster, with each team comprising 2 enumerators. Enumerators were briefed on the study and its objectives and trained to identify eligible participants, obtain informed consent, conduct interviews, and accurately complete data collection tools. After training, all study instruments were pretested in a population outside the study area.

The data collection teams conducted quarterly pregnancy surveillance rounds and visited each household in both the intervention and control clusters to identify pregnant women. The teams were trained to collect information on in- and out-migrations, knowledge and practices related to maternal and newborn health, presence of LHWs at the delivery, and pregnancy outcomes including miscarriages, stillbirths, and neonatal mortality. These teams were blinded to cluster allocations to minimize bias. Data on neonatal and maternal outcomes were collected until the 28th day of life and 40th postpartum day, respectively.

Three independent study intervention teams were used to deliver the maternal and newborn health pack along with instructions on each component of the pack to the study participants in the intervention clusters during the third trimester. Each team consisted of 1 LHW and 2 community health workers. Participants who chose to deliver at home were advised to inform the LHW so that she could attend the birth and conduct timely postnatal visits. The intervention teams also maintained inventory records for the distribution and use of health packs.

#### Control Clusters

In the control clusters, the LHW program continued to function as usual. The LHWs conducted routine household visits while receiving regular refresher training according to the national LHW program.

### Outcomes

The primary outcome of the study was all-cause perinatal mortality, defined as the composite rate of stillbirth (pregnancy loss after 24 weeks per 1000 births per year) and early neonatal mortality (neonatal deaths in the first 7 days per 1000 live births per year). The secondary outcome was all-cause neonatal mortality, defined as neonatal deaths per 1000 live births per year. Late neonatal mortality was defined as deaths from day 8 to day 28 per 1000 live births per year. Additional exploratory outcomes included the rate of miscarriages (pregnancy loss before 24 weeks), community health practices, and postpartum complications.

### Statistical Analysis

Statistical analysis was performed from January to May 2014. Before data entry, all forms were checked for completeness and consistency. Databases and entry screens were developed for data entry using Microsoft Visual FoxPro, version 9.0 (Microsoft Corp). Consistency checks and skips were conducted, and data were entered twice to minimize erroneous data entry. Special arrangements were made to enforce the referential integrity of the database.

The neonatal and perinatal mortality rates during the preceding year were established through data analysis. Descriptive analysis was used to describe the demographic characteristics, socioeconomic characteristics, antenatal care practices, and past pregnancies within the community. The differences in mortalities were assessed using data collected through the baseline survey and surveillance rounds, and cluster-adjusted analysis was conducted to control for variability between clusters.

A modified intention-to-treat analysis was conducted using Stata, version 16 (StataCorp). For analysis of the primary outcome, due to the small number of clusters per group, we opted to follow the method as recommended by Hayes and Moulton.^22^ The perinatal mortality rate for each cluster was calculated, and the logarithm of the cluster-level perinatal mortality rate was used as the independent variable in a linear regression model to estimate the rate ratio associated with the intervention and its 95% CI while accounting for cluster randomization. To adjust the analysis for baseline (preintervention) values, the logarithm of the baseline cluster-level rate was included as a covariate. Furthermore, the regression was weighted based on the number of observations in each cluster. A similar approach was used to analyze the NMR. When analyzing reported practices, we used the svy commands in Stata to account for the clustered nature of the data. A Kaplan-Meier analysis was performed to determine and compare the survival of newborns in the intervention clusters with the survival of newborns in the control group. All *P* values were from 2-sided tests and results were deemed statistically significant at *P* < .05.

## Results

Table 1 presents the overall summary of the demographic characteristics and pregnancy outcomes of the participants (for control group vs intervention group: total number of households, 33 188 vs 34 315; median number of households per cluster, 3092 [IQR, 3018-3467] vs 3469 [IQR, 3019-4075]; total population, 229 155 vs 234 674; mean [SD] number of residents per household, 6.9 [9.5] vs 6.8 [9.6]; number of males per 100 females [sex ratio], 104.2 vs 103.7; mean [SD] number of children <5 years per household, 1.0 [4.2] vs 1.0 [4.3]). The LHW program covered more than 90% of households in both intervention and control clusters. At baseline, the rate of perinatal mortality was 63.8 per 1000 live births, and the rate of neonatal mortality was 51.3 per 1000 live births. Only 46.8% of births (7610 of 16 253) occurred in health care facilities, and skilled birth attendants conducted 46.5% of births (7561 of 16 250). Clean delivery practices were followed in only 7.2% of home deliveries (594 of 8247). All baseline indicators were similar across the intervention and control groups.

**Table 1.** Baseline Population and Household Characteristics

Characteristic	No./total No. (%)	
	Control group (10 clusters)	Intervention group (10 clusters)
Total households, No.	33 188	34 315
Households per cluster, median (IQR)	3092 (3018-3467)	3469 (3019-4075)
Total population, No.	229 155	234 674
Residents per household, mean (SD)	6.9 (9.5)	6.8 (9.6)
Sex ratio, males per 100 females	104.2	103.7
Children <5 y per household, mean (SD)	1.0 (4.2)	1.0 (4.3)
LHWs in post, No.	209	238
LHWs per 10 000 population	9.1	10.1
Houses visited by LHWs	27 813/29 902 (93.0)	28 144/30 299 (92.9)
Educational level of married women and girls aged 15-49 y		
No education	14 221/21 259 (66.9)	14 411/22 314 (64.6)
Primary and middle school	3365/21 259 (15.8)	3946/22 314 (17.7)
Higher secondary school	1678/21 259 (7.9)	2040/22 314 (9.1)
University graduation and above	358/21 259 (1.7)	451/22 314 (2.0)
Religious education	1637/21 259 (7.7)	1466/22 314 (6.6)
Sociodemographic characteristics		
Families owning their own home	29 744/33 186 (89.6)	30 176/34 311 (87.9)
Single-room households	14 845/33 136 (44.8)	14 469/34 256 (42.2)
Households with electricity	30 734/33 057 (93.0)	32 311/34 183 (94.5)
Households with improved drinking water	32 815/33 188 (98.9)	33 599/34 311 (97.9)
Households with improved toilet facility	21 410/33 186 (64.5)	25 064/34 314 (73)
Households using solid fuel for cooking	31 631/33 185 (95.3)	30 404/34 314 (88.6)
Baseline mortality rates		
Stillbirth rate per 1000 births	188/7453 (25.2)	177/7549 (23.4)
Perinatal mortality per 1000 births	495/7453 (66.4)	462/7549 (61.2)
Neonatal mortality per 1000 live births	374/7265 (51.5)	376/7372 (51.0)
Delivery care during last pregnancy		
Facility births	3757/8067 (46.6)	3853/8186 (47.1)
Skilled birth attendants	3753/8066 (46.5)	3808/8184 (46.5)
LHW present at home deliveries	103/4096 (2.5)	88/4205 (2.1)

Abbreviation: LHW, lady health worker.

**Figure 1.**
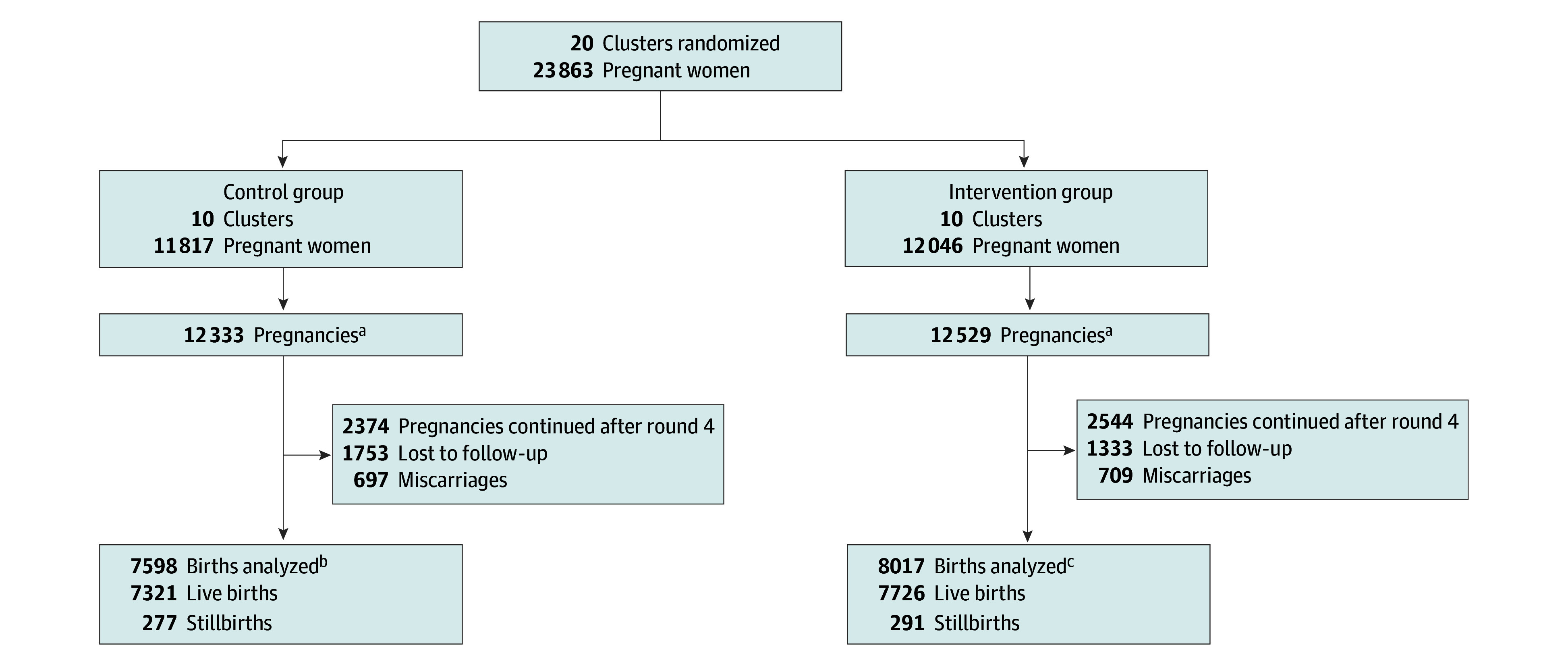
Trial Profile ^a^Number accounts for women who were pregnant more than once during the study period and women who had given birth in the 3 months preceding the first surveillance round. ^b^The 7598 births include 7420 single births and 89 twin births. Pairs of twins included 80 with both live births, 4 with both stillbirths, and 5 with 1 live birth and 1 stillbirth. Overall, there were 7509 deliveries, 604 perinatal deaths, and 382 neonatal deaths. ^c^The 8017 births include 7869 single births and 74 twin births. Pairs of twins included 64 with both live births, 4 with both stillbirths, and 6 with 1 live birth and 1 stillbirth. Overall, there were 7943 deliveries, 540 perinatal deaths, and 303 neonatal deaths.

**Figure 2.**
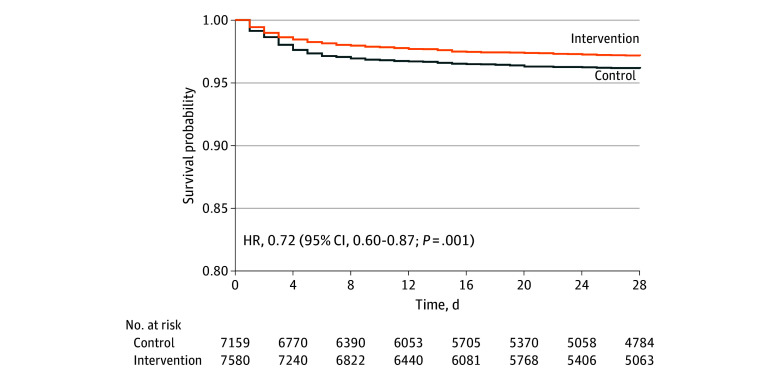
Kaplan-Meier Survival Estimates for Neonatal Mortality HR indicates hazard ratio.

A total of 7943 and 7509 outcomes were captured in the intervention and control clusters, respectively (Figure 1). During the intervention, pregnancy outcomes, including miscarriages, stillbirths, and perinatal and neonatal mortality, were self-reported in quarterly household surveillance visits. The perinatal mortality rate was 67.4 per 1000 live births in the intervention clusters and 79.5 per 1000 live births in the control clusters (rate ratio, 0.86; 95% CI, 0.69-1.08; *P* = .19) (Table 2). The NMR was lower among the intervention clusters vs the control clusters (39.2 per 1000 live births vs 52.2 per 1000 live births; rate ratio, 0.75; 95% CI, 0.58-0.95; *P* = .02). Early NMR was significantly lower among the intervention clusters than the control clusters (32.2 per 1000 live births vs 44.7 per 1000 live births; rate ratio, 0.73; 95% CI, 0.54-0.99; *P* = .04). The hazard of survival was higher among newborns in the intervention clusters (hazard ratio, 0.72; 95% CI, 0.60-0.87; *P* = .001) (Figure 2). An analysis by surveillance round showed similar NMRs among the intervention and control clusters during the first round, with subsequent rounds showing a consistent decrease in NMRs among the intervention clusters (eFigure 2 in Supplement 2). The rates of miscarriages and stillbirths were similar between the intervention and control clusters (Table 2).

**Table 2.** Summary Outcomes

Outcome	Intervention group (10 clusters)	Control group (10 clusters)	Mortality rate ratio (95% CI)^a^	*P* value
All births, No.	8017	7598	NA	NA
Live births identified, No.	7726	7321	NA	NA
Miscarriages				
No.	709	697	NA	NA
Rate per 1000 known pregnancies	82.1	85.4	0.95 (0.77-1.18)	.62
Stillbirths				
No.	291	277	NA	NA
Rate per 1000 total births	36.4	36.6	1.01 (0.80-1.27)	.96
Perinatal mortality				
No.	540	604	NA	NA
Rate per 1000 total births	67.4	79.5	0.86 (0.69-1.08)	.19
Early neonatal mortality				
No.	249	327	NA	NA
Rate per 1000 live births	32.2	44.7	0.73 (0.54-0.99)	.04
Late neonatal mortality				
No.	54	55	NA	NA
Rate per 1000 live births	7.0	7.5	0.84 (0.55-1.28)	.39
Neonatal mortality				
No.	303	382	NA	NA
Rate per 1000 live births	39.2	52.2	0.75 (0.58-0.95)	.02

Abbreviation: NA, not applicable.

^a^ All parameter estimates, 95% CIs, and *P* values were estimated by (weighted) analysis of variance at the cluster level; dependent variable = log (rate); log (baseline neonatal mortality rate) included as a covariate; and the weights used were based on the number of events reported in each cluster.

The numbers of facility births and home births attended by skilled birth attendants were similar among both the intervention and control clusters (Table 3). Lady health workers were more likely to conduct their first postnatal visit within 48 hours in the intervention group than in the control group (risk ratio, 2.20; 95% CI, 1.42-3.42). Clean delivery practices were more common in the intervention group than in the control group (63.2% [2284 of 3616] vs 13.2% [455 of 3458]; risk ratio, 4.80; 95% CI, 3.71-6.22). An analysis by surveillance rounds showed that by the fourth round, 90.2% of participants in the intervention group (920 of 1020) followed clean delivery practices compared with 4.1% (42 of 1027) in the control group. Chlorhexidine use (55.9% [4271 of 7642] vs 0.3% [19 of 7203]; risk ratio, 211.88; 95% CI, 96.93-463.12) and sunflower emollient use (54.9% [4195 of 7642] vs 0.2% [14 of 7203]; risk ratio, 282.43; 95% CI, 106.92-746.07) were significantly more common in the intervention group. Both groups had similar care-seeking behaviors for sick newborns and postpartum complications.

**Table 3.** Maternal, Neonatal, and Child Health Practices Before, During, and After Delivery From Surveillance Rounds 1 to 4

Practice	No./total No. (%)		Risk ratio (95% CI)
	Intervention group (n = 7923)	Control group (n = 7469)	
Women had at least 1 ANC visit by skilled clinician	3203/5130 (62.4)	2719/4430 (61.4)	1.02 (0.93-1.11)
≥4 ANC visits	440/5130 (8.6)	460/4430 (10.4)	0.83 (0.61-1.12)
Women delivered in a health care facility	4249/7923 (53.6)	3973/7469 (53.2)	1.01 (0.88-1.16)
Women delivered by skilled clinician	4725/7923 (59.6)	4166/7469 (55.8)	1.07 (0.96-1.20)
LHW present at home delivery	65/3608 (1.8)	68/3458 (2.0)	0.92 (0.58-1.45)
CDK used for home delivery	2284/3616 (63.2)	455/3458 (13.2)	4.80 (3.71-6.22)
Women visited by LHW within 2 d of delivery	933/7642 (12.2)	399/7203 (5.5)	2.20 (1.42-3.42)
Chlorhexidine used	4271/7642 (55.9)	19/7203 (0.3)	211.88 (96.93-463.12)
Sunflower oil used to massage newborn	4195/7642 (54.9)	14/7203 (0.2)	282.43 (106.92-746.07)
Newborns with reported illness	1572/7642 (20.6)	1571/7203 (21.8)	0.94 (0.73-1.22)
Care seeking for newborns with reported illness	1452/1572 (92.4)	1458/1571 (92.8)	1.00 (0.97-1.02)

Abbreviations: ANC, antenatal care; CDK, clean delivery kit; LHW, lady health worker.

The frequency of sepsis was similar among women in the control (1.1% [9 of 819]) and intervention clusters (1.1% [9 of 815]) (*P* = .99) (eTable 3 in Supplement 2). However, fewer women in the intervention group (46.0% [375 of 815]) had high-grade fever than in the control group (53.6% [439 of 819]) (*P* = .045).

## Discussion

This randomized clinical trial used an integrated community-based intervention package to address neonatal mortality in rural Pakistan. The trial did not demonstrate a significant decrease in stillbirth and perinatal mortality. There is evidence to suggest that fewer antenatal care visits and the absence of a skilled birth attendant are associated with stillbirth and perinatal mortality.^23,24^ Despite the inclusion of a community mobilization component in the present trial, there was no improvement in antenatal and intrapartum care seeking in the intervention group. No additional measures were taken to overcome barriers to accessing health care facilities aside from establishing an emergency transportation system, which may be the reason for the failure of our intervention to increase antenatal and intrapartum health care service use and improve perinatal mortality.

However, this trial did show a reduction in the risk of early and overall neonatal mortality by 27% and 25%, respectively. These results are consistent with previous meta-analyses of trials assessing the effect of community-based interventions on neonatal mortality, demonstrating a 33% reduction in the risk of early neonatal mortality^25^ and a 25% reduction in the risk of overall neonatal mortality.^25,26^ Given that the use of a CDK and chlorhexidine were the only significant improvements in postintervention health care practices, it can be postulated that these were the primary mechanisms for the reduction in neonatal mortality observed in this study. Another important reason for the decrease in neonatal mortality may be the capacity building of health care professionals in the intervention clusters. Several studies have shown that training clinicians in obstetric care can reduce neonatal mortality.^27,28,29,30^ A meta-analysis reported that standardized formal training in newborn resuscitation in low- and middle-income countries can reduce the risk of neonatal mortality by 15%.^31^ It is challenging to attribute the results of the present trial to any specific mechanism due to the multicomponent nature of the study and the possibility of a change in health behaviors that were not captured in this study.

This effectiveness trial offers several crucial insights that should be considered in future scale-up efforts and provides guidance for further research. First, the use of chlorhexidine and CDKs was increased significantly in the intervention clusters. This is important because applying various substances to the umbilical stump, such as oils, creams, antimony, and powders, is a common harmful practice in rural areas of Pakistan,^32^ and substantial evidence supports the use of chlorhexidine and CDKs to reduce neonatal mortality.^33,34,35^ The results of our trial indicate that even in regions with high NMRs, detrimental household health practices, and low levels of care seeking, these interventions can be delivered through existing community health worker programs and improve neonatal survival.

Second, it was possible to closely engage the LHW program for the delivery of the intervention. All LHW sessions were integrated into their regular training, and the LHW supervisors and district health officials consistently monitored LHW activities during the trial. However, the coverage of some components of the intervention remained low, including the presence of LHWs at delivery and postnatal visits within the first 2 days after birth. The operational challenges faced by the LHWs during implementation included difficulty reconciling additional tasks with routine responsibilities, delays in receiving birth notifications, and challenges traveling alone or at night, which demonstrate the limitations of the community outreach program. Last, while there was significant acceptance and uptake of the components of the intervention package at the household level, elements of the intervention package aiming to increase health facility use during pregnancy and delivery did not appear to be as effective, representing an area for further research.

### Limitations

This study has some limitations. There was a possibility of contamination of health messages and information between intervention and control clusters due to the exchange of information between families. To minimize contamination, clusters were defined using existing administrative boundaries, and training sessions were conducted with health care professionals from facilities only in the intervention UCs. Most of the population living in a particular UC was likely to receive care from LHWs and facilities within that UC. The study could not be blinded because of the nature of the intervention. However, separate teams were used for data collection in the intervention and control clusters to minimize the risk of performance bias. All collected data were self-reported, which may have introduced reporting bias due to underreporting of poor pregnancy and newborn outcomes and overreporting of positive newborn care practices among intervention clusters. Moreover, even though data collectors were extensively trained to obtain accurate data, self-reporting may have led to misclassification in early and late neonatal mortality.

## Conclusions

This randomized clinical trial demonstrated a decrease in the NMR and improved household intrapartum and newborn care practices. However, the intervention package did not lead to improvements in the perinatal mortality rate, nor did it increase the frequency of antenatal care visits or facility-based deliveries. These findings underscore that additional efforts are required to devise strategies that can boost care-seeking behavior among pregnant women, increase institutional delivery rates, and decrease perinatal mortality. Nevertheless, the findings from this trial support the scale-up of community-based intervention packages to reduce neonatal mortality in resource-limited regions.

## Notes

Supplement 1.
Trial Protocol

Supplement 2. **eFigure 1.** Map of Tehsil RYK **eTable 1.** Evidence for Each Component of the Intervention Package **eTable 2.** Enhanced Training for Healthcare Providers **eFigure 2.** Neonatal Mortality Rate by Arm and Surveillance Round **eTable 3.** Postpartum Complications
eReferences.

Supplement 3.
Data Sharing Statement

## References

[1] World Health Organization. Levels and trends in child mortality: report 2021: estimates developed by the UN Inter-agency Group for Child Mortality Estimation. December 12, 2021. Accessed May 19, 2023. https://www.who.int/publications/m/item/levels-and-trends-in-child-mortality-report-2021

[2] Sharrow D, Hug L, You D, ; UN Inter-agency Group for Child Mortality Estimation and its Technical Advisory Group. Global, regional, and national trends in under-5 mortality between 1990 and 2019 with scenario-based projections until 2030: a systematic analysis by the UN Inter-agency Group for Child Mortality Estimation. Lancet Glob Health. 2022;10(2):e195-e206. doi:10.1016/S2214-109X(21)00515-5 35063111 PMC8789561

[3] Healthy Newborn Network. Leading causes of neonatal death in Pakistan 2019. 2022. Accessed February 1, 2022. https://www.healthynewbornnetwork.org/country/pakistan/

[4] Every newborn: an executive summary for *The Lancet’s* series. May 2014. Accessed May 12, 2023. https://www.thelancet.com/pb/assets/raw/Lancet/stories/series/everynewborn_exec_summ.pdf

[5] World Health Organization. Maternal mortality. 2019. Accessed February 21, 2022. https://www.who.int/news-room/fact-sheets/detail/maternal-mortality

[6] Asim M, Saleem S, Ahmed ZH, . We won’t go there: barriers to accessing maternal and newborn care in district Thatta, Pakistan. Healthcare (Basel). 2021;9(10):1314. doi:10.3390/healthcare9101314 34682994 PMC8544535

[7] Konje ET, Magoma MTN, Hatfield J, Kuhn S, Sauve RS, Dewey DM. Missed opportunities in antenatal care for improving the health of pregnant women and newborns in Geita district, Northwest Tanzania. BMC Pregnancy Childbirth. 2018;18(1):394. doi:10.1186/s12884-018-2014-8 30290769 PMC6173847

[8] Memon Z, Zaidi S, Riaz A. Residual barriers for utilization of maternal and child health services: community perceptions from rural Pakistan. Glob J Health Sci. 2015;8(7):47-57. doi:10.5539/gjhs.v8n7p47 26925902 PMC4965661

[9] National Institute of Population Studies. Pakistan Demographic Health Survey (PDHS) 2017-18. Accessed May 12, 2023. https://nips.org.pk/publication/pakistan-demographic-health-survey-pdhs-2017-18-main-report

[10] Bhutta ZA, Soofi S, Cousens S, . Improvement of perinatal and newborn care in rural Pakistan through community-based strategies: a cluster-randomised effectiveness trial. Lancet. 2011;377(9763):403-412. doi:10.1016/S0140-6736(10)62274-X 21239052

[11] Jokhio AH, Winter HR, Cheng KK. An intervention involving traditional birth attendants and perinatal and maternal mortality in Pakistan. N Engl J Med. 2005;352(20):2091-2099. doi:10.1056/NEJMsa042830 15901862

[12] Midhet F, Becker S. Impact of community-based interventions on maternal and neonatal health indicators: results from a community randomized trial in rural Balochistan, Pakistan. Reprod Health. 2010;7(1):30. doi:10.1186/1742-4755-7-30 21054870 PMC2993657

[13] Soofi S, Cousens S, Turab A, . Effect of provision of home-based curative health services by public sector health-care providers on neonatal survival: a community-based cluster-randomised trial in rural Pakistan. Lancet Glob Health. 2017;5(8):e796-e806. doi:10.1016/S2214-109X(17)30248-6 28716351 PMC5762815

[14] Mushi D, Mpembeni R, Jahn A. Effectiveness of community based safe motherhood promoters in improving the utilization of obstetric care: the case of Mtwara rural district in Tanzania. BMC Pregnancy Childbirth. 2010;10:14. doi:10.1186/1471-2393-10-14 20359341 PMC2858713

[15] Turan JM, Tesfagiorghis M, Polan ML. Evaluation of a community intervention for promotion of safe motherhood in Eritrea. J Midwifery Womens Health. 2011;56(1):8-17. doi:10.1111/j.1542-2011.2010.00001.x 21323845 PMC3498940

[16] Massavon W, Wilunda C, Nannini M, . Effects of demand-side incentives in improving the utilisation of delivery services in Oyam district in northern Uganda: a quasi-experimental study. BMC Pregnancy Childbirth. 2017;17(1):431. doi:10.1186/s12884-017-1623-y 29258475 PMC5737523

[17] Fleming E, Gaines J, O’Connor K, . Can incentives reduce the barriers to use of antenatal care and delivery services in Kenya?: results of a qualitative inquiry. J Health Care Poor Underserved. 2017;28(1):153-174. doi:10.1353/hpu.2017.0015 28238994 PMC5427715

[18] Bureau of Statistics Punjab. Multiple Indicator Cluster Survey (MICS) Punjab 2017-18, survey findings report. Accessed June 15, 2023. https://bos.punjab.gov.pk/survey_finding_report

[19] Turab A, Pell LG, Bassani DG, . The community-based delivery of an innovative neonatal kit to save newborn lives in rural Pakistan: design of a cluster randomized trial. BMC Pregnancy Childbirth. 2014;14:315. doi:10.1186/1471-2393-14-315 25201572 PMC4177060

[20] World Health Organization. Essential Newborn Care: Report of a Technical Working Group. World Health Organization; 1994.

[21] United Nations Population Fund. Setting standards for emergency obstetric and newborn care. 2014. Accessed March 3, 2022. https://www.unfpa.org/resources/setting-standards-emergency-obstetric-and-newborn-care

[22] Hayes RJ, Moulton LH. Cluster Randomised Trials. 2nd ed. Chapman and Hall/CRC Press; 2017. doi:10.1201/9781584888178

[23] Ota E, da Silva Lopes K, Middleton P, . Antenatal interventions for preventing stillbirth, fetal loss and perinatal death: an overview of Cochrane systematic reviews. Cochrane Database Syst Rev. 2020;12(12):CD009599. 33336827 10.1002/14651858.CD009599.pub2 PMC8078228

[24] Bhutta ZA, Yakoob MY, Lawn JE, ; Lancet’s Stillbirths Series steering committee. Stillbirths: what difference can we make and at what cost? Lancet. 2011;377(9776):1523-1538. doi:10.1016/S0140-6736(10)62269-6 21496906

[25] Lassi ZS, Bhutta ZA. Community-based intervention packages for reducing maternal and neonatal morbidity and mortality and improving neonatal outcomes. Cochrane Database Syst Rev. 2015;2015(3):CD007754. doi:10.1002/14651858.CD007754.pub3 25803792 PMC8498021

[26] Hanson C, Kujala S, Waiswa P, Marchant T, Schellenberg J. Community-based approaches for neonatal survival: meta-analyses of randomized trial data. Bull World Health Organ. 2017;95(6):453-464C. doi:10.2471/BLT.16.175844 28603312 PMC5463806

[27] Carlo WA, Goudar SS, Jehan I, ; First Breath Study Group. Newborn-care training and perinatal mortality in developing countries. N Engl J Med. 2010;362(7):614-623. doi:10.1056/NEJMsa0806033 20164485 PMC3565382

[28] Sorensen BL, Rasch V, Massawe S, Nyakina J, Elsass P, Nielsen BB. Advanced Life Support in Obstetrics (ALSO) and post-partum hemorrhage: a prospective intervention study in Tanzania. Acta Obstet Gynecol Scand. 2011;90(6):609-614. doi:10.1111/j.1600-0412.2011.01115.x 21388368

[29] Msemo G, Massawe A, Mmbando D, . Newborn mortality and fresh stillbirth rates in Tanzania after Helping Babies Breathe training. Pediatrics. 2013;131(2):e353-e360. doi:10.1542/peds.2012-1795 23339223

[30] Walker DM, Holme F, Zelek ST, . A process evaluation of PRONTO simulation training for obstetric and neonatal emergency response teams in Guatemala. BMC Med Educ. 2015;15:117. doi:10.1186/s12909-015-0401-7 26206373 PMC4513701

[31] Pammi M, Dempsey EM, Ryan CA, Barrington KJ. Newborn resuscitation training programmes reduce early neonatal mortality. Neonatology. 2016;110(3):210-224. doi:10.1159/000443875 27222260

[32] Coffey PS, Brown SC. Umbilical cord-care practices in low- and middle-income countries: a systematic review. BMC Pregnancy Childbirth. 2017;17(1):68. doi:10.1186/s12884-017-1250-7 28219420 PMC5319165

[33] Seward N, Osrin D, Li L, . Association between clean delivery kit use, clean delivery practices, and neonatal survival: pooled analysis of data from three sites in South Asia. PLoS Med. 2012;9(2):e1001180. doi:10.1371/journal.pmed.1001180 22389634 PMC3289606

[34] Park JH, Hamer DH, Mbewe R, . Components of clean delivery kits and newborn mortality in the Zambia Chlorhexidine Application Trial (ZamCAT): an observational study. PLoS Med. 2021;18(5):e1003610. doi:10.1371/journal.pmed.1003610 33951036 PMC8133479

[35] Shariff JA, Lee KC, Leyton A, Abdalal S. Neonatal mortality and topical application of chlorhexidine on umbilical cord stump: a meta-analysis of randomized control trials. Public Health. 2016;139:27-35. doi:10.1016/j.puhe.2016.05.006 27311991

