# Supplementary material for: Effect of Maternal and Newborn Care Service Package on Perinatal and Newborn Mortality: A Cluster Randomized Clinical Trial
Source: JAMA Netw Open. 2024 Feb 19;7(2):e2356609. doi: 10.1001/jamanetworkopen.2023.56609 (PMC10877450; doi:10.1001/jamanetworkopen.2023.56609)
Supplement: Supplement 1. — Trial Protocol [file jamanetwopen-e2356609-s001.pdf]

A Research Proposal by the Women & Child Health Division,
Aga Khan University, Karachi, Pakistan

Research Proposal:
Improved accessibility of EmONC services for Maternal and Newborn Health: a Community Based Project

Submitted to

Research and Advocacy Funds, RAF

Senior Consultant

Prof Zulfiqar A Bhutta

Chair, Women & Child Health Division

Aga Khan University

Principal Investigator

Dr. Sajid Bashir Soofi

Assistant Professor

Women & Child Health Division

Aga Khan University

Co-Investigators

Zohra Lassi

Senior Instructor

Women & Child Health Division

Aga Khan University

Dr. Atif Habib

Senior Instructor

Women & Child Health Division

Aga Khan University

Dr. Shabina Ariff

Assistant Professor

Women & Child Health Division

Aga Khan University

Executive Summary

Deaths during birth are particularly critical to address as these occur because of complications during childbirth and leads to emergency situation with a slim window of time to intervene. Every year an estimated 3.6 million newborns and 360,000 mothers die globally. Of these, maternal health complication contribute to 1.5 million of neonatal deaths in the first week of life and 1.4 million stillborn neonates, suggesting a major gap of intervention subsists around childbirth and in the early postnatal period, a time when mothers and babies are most at risk. While many factors contribute to maternal and neonatal deaths, one of the most effective means of solving this problem requires effective preventive measures or treatment provided rapidly to women and newborns, often at home or in primary health care settings.

It is often addressed that these overwhelming mortalities and morbidities are closely linked with a number of interrelated delays that prevent a pregnant women from accessing the health care she needs. Each delay is closely related to services, logistics, facilities and conditions, which are important elements for their health. These delays are: 1) delay in seeking appropriate medical help for an obstetric emergency or neonatal complication for reasons of cost, lack of recognition of an emergency, poor education, lack of access to information and gender inequality; 2) delay in reaching an appropriate facility for reasons of distance, infrastructure and transport; 3) delay in receiving adequate care when a facility is reached because there are shortages in staff, their competency, or due to unavailability of required medical facilities and equipment. As a result, many preventable maternal deaths occur, most often in resource poor settings, where births are home-based and in the event of complications the woman is unable to access the required care in time.

While many proven, cost-effective ways to save the lives of mothers, and newborns exist, they are not always available to those who need them most. There is bulk of literature that has identified number of interventions that can improve maternal and newborn health. The lancet maternal and neonatal survival series emphasized the model of "Basic essential obstetric care" as one of the most feasible and effective strategies to reduce maternal mortalities.

For the reasons above, it is essential to create demand of uptake of services and strengthen primary health care infrastructure at the community level, and improve the liaison of LHWs/TBAs/CMWs with local and district health system for early and timely referral of complicated cases and sick newborns. While several previous studies from Pakistan have documented the beneficial impact of community-based interventions in improving maternal and new-born health, further evidence is required to assess the effectiveness of community-based interventions that can increase the uptake of EmONC services and reduce the delays that are responsible for poor maternal and new-born health. Context-specific evidence is also needed on the appropriate mix of interventions, their delivery strategies, task shifting and sharing options, functional link and assessment in the primary health care, and complementary health systems and community support and demand mechanisms.

Therefore, the main target audience and beneficiary of this project is the women which usually do not have a say in the decision making and cannot have choices for her to opt for a better

treatment. The women usually depend upon the decisions being made by the husbands and elders as the society is male dominant. We have attempted to take care of this factor to maximize the uptake of intervention and services by developing the community support groups.

The specific objectives of this study are:

- To conduct an in-depth analysis of maternal and neonatal health seeking patterns and behaviors of the target population and care provision at health facilities for understanding the context and requirements for improved EmONC service delivery.
- To mobilize community for creation of demand for improved MNH services and practices through community mobilization.
- To train and implement integrated EmONC package for community-level health care providers (CMWs/TBAs and LHWs) to provide antenatal, natal and postnatal care services, and recognize and refer complicated pregnancy and childbirth cases and sick newborns to health facilities.
- To strengthen and improve the quality of care at health facilities in providing EmONC services through capacity building of health care providers.
- To create linkages between the cadre of health care providers thereby ensuring their presence at the time of delivery and in case of referrals
- To utilize communication technology (in the form of health text messages) for community awareness and mobilization and to motivate LHWs to be present at deliveries.

A cluster randomized controlled trial with a mix of formative (including quantitative and qualitative) research will be conducted in Tehsil Rahim Yar Khan of District Rahim Yar Khan. An initial baseline household survey will be conducted before randomization of intervention and cluster arms to understand the typology and socio-demographic characteristics of area under study. Furthermore, focus group discussion (on women of reproductive age, mother-in-law and male members of society) will be conducted to assess the knowledge attitude and practices of community and to understand their cultural values. Also in-depth interviews with healthcare staff and health managers will be undertaken to assess the structural, operational and resource allocation re-adjustment required to improve the health status.

After this formative research, implementation research will be undertaken to implement EmONC package to improve access to services required during emergency situation. This will consist of building community support groups to create awareness in the community and at the household level. In these support groups, funds for emergency transportation will be established.

The LHWs, CMWs and TBAs in the interventions arms will be given enhanced training on early recognition of high risk pregnancies, deliveries and sick newborns. This will enhance their skills in identifying the need for referral. They will also facilitate transfer to an EmONC facility by identifying and arranging methods of transportation. They would be responsible to stabilize and manage woman/newborn prior to transfer; and communicate with hospital staff for timely and effective management. Furthermore, LHW and CMWs will be equipped with a communication system (mobile/cell phone) to link her with the community and health facility. Families of pregnant women and newborn babies will be given the number of their area assigned

LHW/CMW. In case of emergency, LHW will prepare health staff at the concerned health facility for receiving case she has referred, hence a functional referral link (defined above) will be enhanced between the components of PHC (household/ family, first level health care providers and first level facility), and with EmONC facilities to ensure the required continuity of care throughout pregnancy, childbirth and postnatal care.

On the other hand, health care staff at BHU/RHC will be given training on basic EmONC and staff at the THQ and DHG will be provided training on comprehensive EmONC. We will also institute an emergency tray at all the health care facilities existing in the study area to prepare and equip them with essential lifesaving drugs (such as adrenalin, hydrocortisone and crystalloids) and equipment (such as laryngoscope and endotracheal tubes) to deal acute maternal and neonatal emergencies.

A baseline survey at household and health care facilities will be conducted in addition to interviews and focus group discussions. Also routine record keeping will be processed during the study period. Ongoing surveillance will be conducted to compare the effectiveness of EmONC package. Data collected will be cross checked at all levels (field to central unit) and analysis will be conducted separately for qualitative and quantitative aspects.

Background

1. Global situation on maternal and newborn health

Deaths during birth are particularly critical to address as these occur because of complications during childbirth and leads to emergency situation with a slim window of time to intervene. Every year an estimated 3.6 million newborns and 360,000 mothers die globally.[1] Of these, maternal health complication contribute to 1.5 million of neonatal deaths in the first week of life and 1.4 million stillborn neonates, suggesting a major gap of intervention subsists around childbirth and in the early postnatal period, a time when mothers and babies are most at risk.[1] Even with improvements in certain areas, deaths of children under five years and the risk of mortality in pregnancy or childbirth remain incongruously high in sub-Saharan Africa and Southern Asia.[2] While many factors contribute to maternal and neonatal deaths, one of the most effective means of solving this problem requires effective preventive measures or treatment provided rapidly to women and newborns, often at home or in primary health care settings.

It is often addressed that these overwhelming mortalities and morbidities are closely linked with a number of interrelated delays that prevent a pregnant women from accessing the health care she needs. Each delay is closely related to services, logistics, facilities and conditions, which are important elements for their health (**Figure 1**). These delays are: 1) delay in seeking appropriate medical help for an obstetric emergency or neonatal complication for reasons of cost, lack of recognition of an emergency, poor education, lack of access to information and gender inequality; 2) delay in reaching an appropriate facility for reasons of distance, infrastructure and transport; 3) delay in receiving adequate care when a facility is reached because there are shortages in staff, their competency, or due to unavailability of required medical facilities and equipment. As a result, many preventable maternal deaths occur, most often in resource poor

settings, where births are home-based and in the event of complications the woman is unable to access the required care in time.[3]

Figure 1: The 3 delays model of maternal mortality

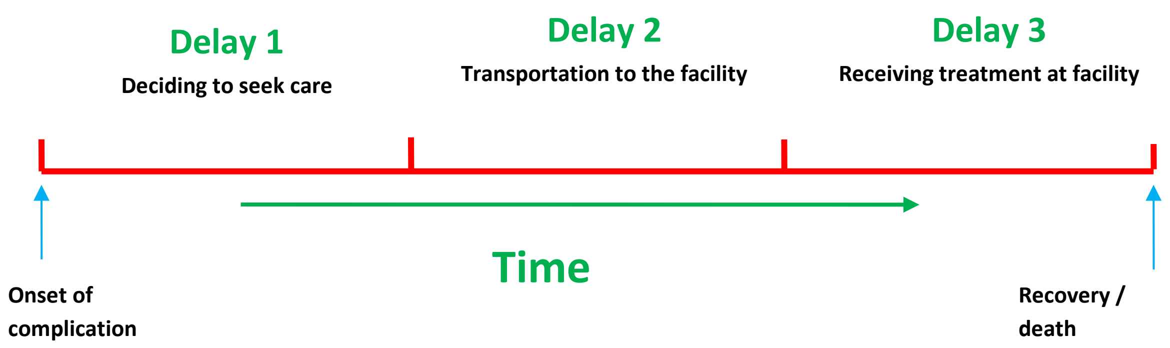

Furthermore, the health of neonates is intimately related with the health of the mother and a large number of newborns deaths occur on the first day of life due to complications during childbirth. It is argued that addressing these deaths requires combined packages that address the problem at each level of delay. The most competent and effective strategy is the provision of care to the mother and the newborn through a "continuum of care"[4], which commences when women are young, begins well before they conceive, helps prevent unintentional pregnancies and persists through antenatal, natal and post natal period into their child's early years, and which incorporates household, community and health care system.[4] This approach helps to avoid the disparity between maternal and child health issues and health services delivery.[5]

2. Maternal and neonatal health in Pakistan

The health status of women in Pakistan is even poorer in contrast to other neighboring countries in Asia. In recent reports by UNICEF and PDHS, the reported maternal mortality ratio is found to 276 per 100,000 live births.[6] Furthermore, the early mortality or morbidity of a woman is overwhelming and also affects the health of her children. The situation for the under-1 year mortality is 78 and the neonatal mortality is 57. It is estimated that annually 216,000 Pakistani newborns die before they reach their first month of age, which represents 58% of total deaths of children under five. These deaths are more prevalent in rural areas particularly among poor families as compare to urban areas. The maternal mortality in urban areas is 204 compared to 336 per 100,000 live births in Pakistan. Similarly neonatal mortality ratio in urban areas is 48 per 1000 live births compared to 55 per 1000 live births in rural areas. However, NMR in highest wealth quintile in 38 compared to 63 per 1000 live births in lowest wealth quintile.

3. Health care infrastructure in Pakistan

Pakistan has an impressive infrastructure for primary health care with a network of basic health units (BHU) and rural health centers (RHC)[7] (**Figure 2**). While the RHCs and BHUs are staffed by qualified medical and nursing staff, health care at the community level is largely supported by a

large number of trained cadres of lady health workers (LHW) and newly deployed community midwives (CMWs). Although CMWs are trained in the identification of complicated pregnancy and its management, but very few have been deployed so far, and many districts do not have any CMWs. Also the services for basic or comprehensive emergency obstetric or newborn care at the level of BHUs and RHCs are questionable (in terms of quality). Similarly, there is very little emphasis in the current training program of LHWs as well as primary care LHVs and physicians on EmONC services. The latter is important as available evidence indicates that despite impressive reduction in infant mortality rates in certain areas such as the northern areas, perinatal mortality still remains distressingly high (AKHSP Northern Areas Annual Report 2000). According to PDHS 2006-07, NMR is high is Punjab (58 per 1000 live births) compared to 30 per 1000 live births in Baluchistan.

There is considerable potential for training of LHWs and CMWs in Pakistan, in order to improve maternal and newborn care. Currently almost 102,000 LHWs are in place, and while they are not directly involved in deliveries, these LHWs are supposed to function in close liaison with BHU and RHC staff in providing antenatal care, contraceptive advice, growth monitoring and immunization services.

A largely ignored cadre of primary care workers in Pakistan is the semi-trained and traditional birth attendants (TBAs). This large group of individuals is responsible for the vast majority of births in rural settings and a large proportion of births even in urban areas (**Figure 3**). The TBA (Dai) has been recognized as an important resource person in the quest for improving reproductive health delivery, both in relation to maternal as well as newborn care, particularly in those areas where no formal health care outreach services are available.

Figure 2: Health care infrastructure in Pakistan

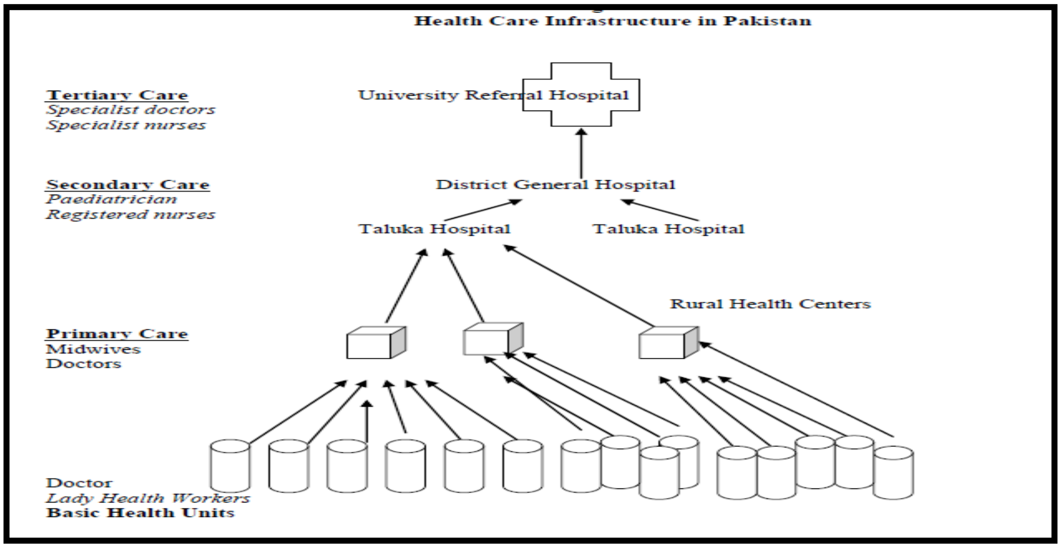

Figure 3: Who delivers?

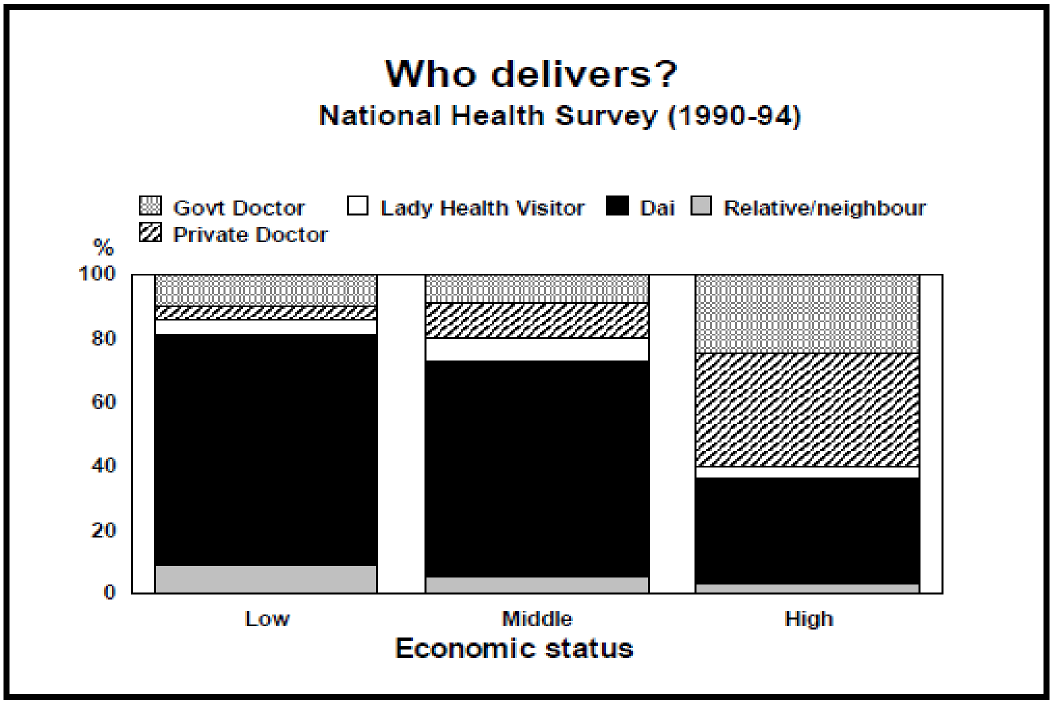

Government of Pakistan has recently started investing in training and deploying community midwives in every district of Pakistan, with the aim of extending coverage by skilled birth attendants to 70% of home deliveries and to address a huge gap of skilled birth attendance utilization at birth (the first batch of CMWs graduated from training in 2010). CMWs are expected to be able to address issues critical and emergency situations such as complication during labor, birth asphyxia, prematurity related problems and neonatal infections. However, it is unclear how many have been trained and deployed in each district so far. In addition, it is too early to know whether, upon successful completion of training, the CMWs will be willing to serve in the rural areas and provide care at household level. India's experience, for example, has not been very encouraging. Bang et al. (2005)[8] report that a full-time paid cadre, called 'an auxiliary nurse-midwife', (one midwife covering five thousand population) created in the early 1990s all over India, was present only in 15% of home deliveries. They suggest that a community health worker who is from the same village is more likely to attend home deliveries. There is also evidence from Sindh to indicate that Traditional Birth Attendants (TBAs) can link satisfactorily with health system staff, especially with the LHWs.[9]

4. Case for Improved EmONC facilities in Pakistan

There is bulk of literature that has identified number of interventions that can improve maternal and newborn health. The lancet maternal and neonatal survival series[10, 11] emphasized the model of "Basic essential obstetric care" as one of the most feasible and effective strategies to reduce maternal mortalities.[10] The 2008 Lancet Alma-Ata series emphasized skilled care at facility levels for saving maternal lives and scaling up of community and household care for improving newborn and child survival. The series identified 37 key promotional, preventive, and treatment interventions and strategies for delivery in primary health care.[12] An estimate conducted by the World Bank conclude that about 74% of maternal deaths could be avoided if all women accessed

interventions that address complications of pregnancy and childbirth, especially emergency obstetric care.[13] The interventions set out for child survival by the Bellagio study group reviewed and presented the existing evidence based interventions to reduce neonatal mortality. It is estimated that universal coverage percent (proven neonatal interventions) of established maternal and neonatal health interventions could prevent a large number of mortalities.

While many proven, cost-effective ways to save the lives of mothers, and newborns exist, they are not always available to those who need them most. Poor, who are far behind in accessing formal education, usually lack awareness on health issues, doesn't have capacity to reach to health service and top of all lack money to pay for services. Therefore, simple interventions that can target such areas have potential to improve health status.

Historically overlooked by both safe motherhood and child survival policies and programs, newborns continue to lack access to cost-effective lifesaving interventions. The Bellagio Study Group on Child Survival estimates that universal coverage (99%) of 16 proven newborn health interventions could avert up to 72 percent of all newborn deaths. These include interventions such as tetanus toxoid immunization, skilled access to emergency obstetric care, immediate and exclusive breastfeeding, drying and keeping the newborn warm, and if needed, resuscitation, care of low birth weight infants, and treatment of infection. Apart from direct provision of interventions, research has shown that interventions that involve community mobilization and empowerment, and include counseling to mothers on topics related to birth and newborn care preparedness for bringing behavior change can reduce total neonatal deaths by 21% and early neonatal deaths by 24%.[14] On the other hand, improvement of women's access to antenatal, intrapartum and postnatal care with training cadre of community workers, and traditional birth attendant can reduce maternal morbidities by 25% and further improve total neonatal mortalities by 23%.[14] Additional strategies against rising toll of deaths necessitate development in health systems and primary health care to ensure availability of skilled attendance at all levels and access to 24-hour emergency obstetric care. Since many opportunities to optimize birth outcomes occur at the time of birth, therefore it is also important to improve access to skilled birth attendance and emergency obstetric and newborn care that can deliver parenteral administration of drugs, blood transfusion, and surgical interventions when required. Recent reviews and evidence from community based effectiveness studies show that large proportion of these deaths can be saved with the presence of functional referral system.[15, 1617]

The experience of Hala perinatal trial (both during the pilot and scale-up phase)[18, 19] in which intervention package was delivered through community-based LHWs and TBAs indicates that it is possible to achieve improvements in household care seeking behaviors with an overall 25-30% reduction in perinatal and neonatal mortality. The trial was also associated with significant improvement in care seeking and skilled attendance in facility settings. However, notwithstanding the observed improvements in skilled attendance, clear needs have been identified for further improvement in facility-based care, and demand creation for institutional care especially by creating linkages with health facilities through preparing community for building funds for emergency transportation.

Justification of the Study

Most maternal and newborn deaths occur around labor, delivery and immediate postpartum period. An overwhelming 75% the neonatal deaths occur in the first week of life, more than 25% in the first 24 hours after birth. Twenty three percent (23%) of neonatal death and 26% of stillbirths are due to intrapartum events. Furthermore, between 11-17% of maternal deaths happen during the childbirth itself, and 45% of postpartum maternal deaths occur during the first 24 hours and more than two thirds in the first week.

Majority of maternal and perinatal deaths are preventable through proper understanding, recognizing, screening, and management of complications. However, services are fragmented due to a host of interrelated technical, operational and political challenges. These are related to barriers on both the supply (poor services, inadequate health staff) and demand (lack of awareness, poor access to services and inability to pay for services) of the health care systems. While striving to overcome the obstacles in the larger health systems context, alternate and practical approaches are needed to implement evidence-based interventions to reduce the maternal and perinatal mortality.

Therefore, the main target audience and beneficiary of this project is the women who usually do not have a say in the decision making and cannot have choices for her to opt for a better treatment. The women usually depend upon the decisions being made by the husbands and elders as the society is male dominant. We have attempted to take care of this factor to maximize the uptake of intervention and services by developing the community support groups.

For the reasons above, it is essential to create demand of uptake of services and strengthen primary health care infrastructure at the community level, and improve the liaison of LHWs/TBAs/CMWs with local and district health system for early and timely referral of complicated cases and sick new-borns. While several previous studies from Pakistan have documented the beneficial impact of community-based interventions in improving maternal and new-born health,[20-22] further evidence is required to assess the effectiveness of community-based interventions that can increase the uptake of services and reduce the delays that are responsible for poor maternal and new-born health. Context-specific evidence is also needed on the appropriate mix of interventions, their delivery strategies, task shifting and sharing options, functional link and assessment in the primary health care, and complementary health systems and community support and demand mechanisms.

The Maternal, Newborn and Child Health Program (MNCH) of the MoH, Pakistan is committed towards improving the maternal, newborn and child health in Pakistan in line with MDGs 4 & 5, with support and concerted efforts of the government and donors. This RAF is to support advance and consolidate the GoP-MNCH program strategies to strengthen the MNH service delivery through effective evidence.

Research Questions

Does an integrated EmONC package (community mobilization, training of community-based health care providers) reduce perinatal and neonatal mortality?

Objectives:

- To conduct an in-depth analysis of maternal and neonatal health seeking patterns and behaviors of the target population and care provision at health facilities for understanding the context and requirements for improved EmONC service delivery.
- To mobilize community for creation of demand for improved MNH services and practices through community mobilization.
- To train and implement integrated EmONC package for community-level health care providers (CMWs/TBAs and LHWs) to provide antenatal, natal and postnatal care services, and recognize and refer complicated pregnancy and childbirth cases and sick newborns to health facilities.
- To strengthen and improve the quality of care at health facilities in providing EmONC services through capacity building of health care providers.
- To create linkages between the cadre of health care providers thereby ensuring their presence at the time of delivery and in case of referrals
- To utilize communication technology (in the form of health text messages) for community awareness and mobilization and to motivate LHWs to be present at deliveries.

Methodology

Study design

It is a cluster randomized controlled trial. A formative research will follow a mixed-methods approach to achieve the objectives of the study. Mixed method research that contains both qualitative and quantitative approaches is commonly used to explore the context of an issue and combines the strengths and lessens the weaknesses in both approaches within a single study.

We are proposing a mixed method design in which the qualitative research will precede the quantitative.

Qualitative component	Quantitative component
1. Focus group discussion (FGDs)<br>2. In-depth Interviews (IDIs)	3. KAP survey<br>4. Health facility surveys and audits<br>5. Implementation research

For the qualitative component focus group discussions (FGDs) and in-depth interviews (IDIs) will be conducted on different target groups.

For the quantitative component a baseline knowledge attitude & practices (KAP) survey will be conducted on 10% of the population. We will also conduct assessments and audits of all the health facilities within the target areas. The findings of IDIs, FGDs and KAP survey would give an insight on the acceptability of proposed package of care. Also based on findings we will devise the best way to implement the proposed EmONC package in community.

Study Site

The Study will be conducted in Tehsil Rahim Yar Khan of District Rahim Yar Khan (RYK). The District Rahim Yar Khan (population~4,580,000) is one of the largest districts of southern Punjab, consisting of four sub-divisions (Tehsils) i.e. Sadiqabad, Rahim Yar Khan, Khanpur and Liaqatpur.

District Rahim Yar khan has been selected as the target district not only because of alarming Maternal and Child health indicators but because of the presence and strong community connection of Women and Child Health division of Aga Khan University. The University has a project office at Rahim Yar Khan from where major Flood Relief Activities were done; another major nutrition intervention project was carried out in the district through the involvement of Lady Health workers and local government. Hence these contributions and connections with the LHW program, local government and community provide a feasible environment for the Aga Khan University to undertake this project.

GENERAL DEMOGRAPHIC INFORMATION OF DISTRICT RAHIM YAR KHAN

**Indicators**	
Population	4578538
The land area of the district	11880 sq.km
Average Annual Growth Rate	3.2%
Population density	385/square KM
Infant < 1 yr (expected in 2010)	123621
Children < 5 yr(expected in 2010)	613524
LHWs (expected in 2010)	1851
Women in child bearing age (expected in 2010)	1007278
Married child bearing age women (expected in 2010)	732566
Expected pregnancies (expected in 2010)	155670

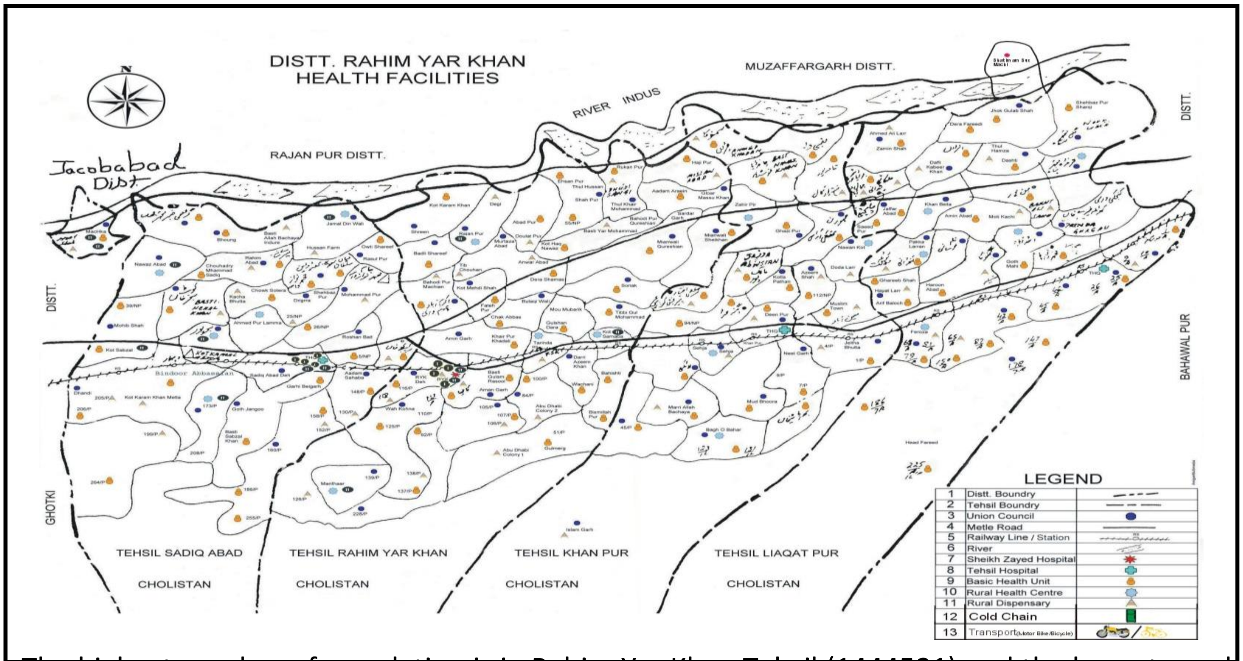

The highest number of population is in Rahim Yar Khan Tehsil (1444531) and the lowest number of population is in Khanpur (995728). The rural population is more (82%) than the urban (18%) population. In terms of population demographic, there are more females (52%) compared to males (48%) in the district and almost half majorities? (44%) of people are below 15 years of age.

Tehsil wise distribution

Tehsil	Number Of UC	Population

Khanpur	28	995728
Liaqatpur	25	1005625
Rahim Yar Khan	40	1444531
Sadiqabad	29	1132654

	DHQ	THQ	RHC	BHU	Dispensaries
District RYK	01	04	19	104	56
Tehsil RYK		1	5	30	16

Health Indicators

Indicators	
Infant Mortality Rate	98/1000 live births
Under 5 mortality Rate	148/1000 live births
Maternal mortality Ratio	103/100,000 live births

Sources: MICS 2007-08 and EDOH Office Rahim Yar Khan

Research snapshot

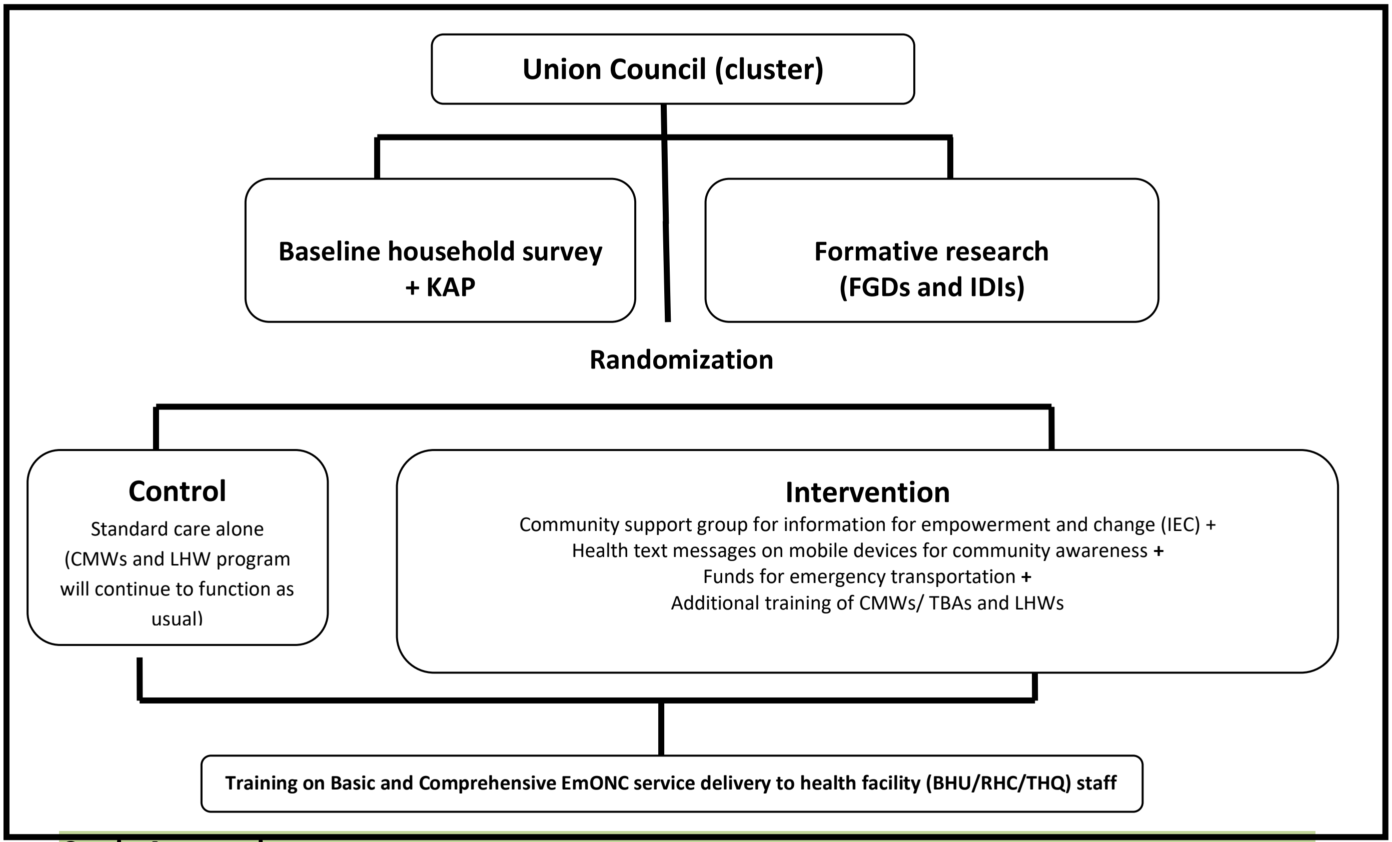

Study Approaches

Qualitative Component

In order to conduct a comprehensive research in the community and to implement suitable interventions, it is imperative that accurate information on the etiology of maternal and neonatal morbidity and mortality is available. This, however, is difficult as much of the information on mortality/morbidity trends and associated outcomes is only available from hospital-based cohorts. It is therefore, crucial to collate such information from community. Also, in order to

incorporate culturally sensitive and logistically appropriate factors, we need to understand the target population. Therefore, this initial phase of qualitative research will help us in understanding the embedded problems and also an in-depth evaluation of knowledge, attitude and practices of families and primary care providers (TBAs, LHWs, LHVs and BHU/RHC staff) will assist us in understanding the health situation in study area. These issues are with regards to

- Common causes of maternal morbidity in pregnancy
- Maternal nutrition and dietary patterns during pregnancy
- Determinants of care seeking behavior during pregnancy and labor
    - Domiciliary versus hospital births
    - Selection of care givers by families
    - Reasons for referral by primary care givers
- Knowledge, Perception and recognition of danger signs during pregnancy and child birth by families and care givers
- Immediate and early newborn care practices (colostrum and prelacteal feeding, breastfeeding, cord care, temperature regulation, swaddling, skin care etc.)
- Concepts of early neonatal morbidity and health seeking behavior e.g.
    - Risk factors for birth asphyxia
    - Low birth weight and temperature regulation)
    - Neonatal infection (local and generalized)
    - Seizures or spasms
    - Respiratory problems

1. Sampling Methodology:

We are proposing purposive sampling according to the framework in following tables to ensure to include population from poor and marginalized groups received representation. Sample size for qualitative research usually depends on the resources, time available, and a study's objectives. Purposive sample sizes are often drawn on the extent of level of data saturation. It is the point in data collection when new data no longer bring additional insights to the research questions or objectives. Purposive sampling is most successful when data review and analysis are done in conjunction with data collection. The sample size for FGDs and IDIs are as follows:

Sample size for Focus Group Discussions& IDIs (Tehsil Rahim Yar Khan):

Respondents	Research Methods	No. of Interviews / Discussions
Recently Delivered Women	IDIs	06
Currently Pregnant Women	IDIs	06
Lady Health Workers	FGDs	02
Traditional Birth Attendants	FGDs	02
Community Midwives	IDIs	06
Community Leaders	IDIs	04
Male decision makers in HH	FGDs	02
Female decision makers in HH	FGDs	02
Executive District Officer (Health)	IDIs	01
District Coordinator (National Program)	IDIs	01
CMW Coordinator	IDIs	01

General Physicians	IDIs	02
Pediatricians	IDIs	02
Gynecologists	IDIs	02

2. Sample Size Calculation for KAP Survey (Tehsil RYK):

We will also conduct a KAP survey during the initial baseline survey which will be done in the target study area. We will implement the cluster sampling methodology for KAP survey. For the calculation of sample for the KAP survey we have selected the household as a Primary Sampling Unit (PSU) for our survey and we will select one women of reproductive age from each household. Firstly we will calculate the sample size required for the survey and then this sample size will be distributed through cluster sampling in target union councils (household will be selected systematically i.e. every k^{th} from random starting point). The overall calculation for the sample size is as follows. Using the formula for calculating sample size:

$$N=Z^2 (p) (q)/d^2 X DE$$

Assuming

- **Z**=1.96. (Confidence interval)
- **p** and **q**=(1-p) = 0.5 by assuming a 50/50 split in responses across questions. (Refers to the proportion of the population with the attribute we are looking).
- **d = 5%** refers to the desired precision of the estimate.
- **DE** = 2 (Design effect)

$$N= (1.96) (.5)(.5)/0.05^2 X 2 = 768$$

Quantitative approach:

Cluster randomized control trial will be used to evaluate the impact of EmONC package for maternal and neonatal health care delivery through community level health care providers. Clusters will be identified and matched on population size, socio-economic status (including structure, electricity, drinking water with HH density), birth/death rates, and number of functional LHWs and CMWs. Cluster Paired randomization will be done to allocate one group (intervention/control) to each cluster and all the LHWs in one union council will be assigned to one group to avoid any sort of contamination.

Sample Size Calculation: We are considering Union Council as a cluster. Each Union council serves approximately 15000 - 20000 population and usually contains a Basic health Unit which provides basic facilities of antenatal, natal and post natal care.

- We assume that the average cluster size is 15000 and considering a crude annual birth rate of 20 per 1000 and the estimated average perinatal mortality rate of 60/1000 with a coefficient of variation between clusters of 0.125 and to detect a 25% reduction in the mortality rates, we would require **20 Clusters** in total. Ten clusters per arm to provide the study with a power of 90% over a three-year intervention period.

EmONC Package:

On the basis global and local literature available around the maternal and neonatal care and survival, a comprehensive EmONC package is developed to be rolled out in the target areas of District RYK.

Operational definition of Emergency Obstetric and Newborn Care

Basic EmOC:

- Parenteral antibiotics
- Parenteral oxytocics
- Parenteral anti-convulsants and anti-hypertensives
- Manual removal of placenta
- Removal of retained products (MVA)
- Assisted vaginal delivery (forceps, vacuum extraction)

Comprehensive EmOC:

- All 6 basic functions plus:
- Blood transfusion
- Cesarean section

Emergency Obstetric and Newborn Care (EmONC) (in addition to the above)

- Neonatal resuscitation;
- Hypothermia Management (re-warming);
- Antibiotics for neonatal sepsis (injectable and oral);
- Essential newborn care.

Demand side interventions

a) ***Community Mobilization/Community Supports Groups:***

To create awareness in the community and at the household level in intervention clusters, information for empowerment and change (IEC) strategy for women and their husbands through female and male supports groups will be formed/ strengthened. The LHWs will identify and train community facilitators. Female activist will form female support groups and male activists will form male support groups in the LHW catchment area. These activists will enter and raise awareness about MNH and will work with community to identify and invite those most likely to be affected such as poor and marginalized.

These facilitators will be trained on disseminating awareness on antenatal care, identification of danger signs related to pregnancy and recognition for high-risk pregnancies and births (these include maternal malnutrition, illness, short stature, previous perinatal deaths etc.), birth preparedness (skilled birth attendant, facility), essential & immediate newborn care and recognition of danger signs and management low birth weight and sepsis with early and appropriate referral. An emergency fund will be established in community support groups for active referral and transportation of the complicated cases for the provision of EmONC. These community groups will aid in establishing funds from the local resources and families will be asked to contribute whatever is in their capacity, and utilization of these funds will be monitored by the person from community in charge for this activity so that each family benefit equally.

The LHWs and the family members in the intervention community will also be sent informational/educational messages on their mobile phones to highlight the importance of maternal and newborn health. The contact numbers will be collected during the base line survey.

1. Proposed EmONC Package

	Interventions				Actions
For intervention arm	**Community Mobilization**	Advocacy session and community mobilization for **women of reproductive age, pregnant women, mothers, mother-in-laws, husband, community gate keepers, and elders**<br>Mobile messages in Urdu for family and LHW on important events in pregnancy ,delivery and postnatally for f maternal and newborn health<br>Funds for emergency transportation			Community support groups will be formed which will help to develop linkages between the communities and health facilities and funds for emergency transport
	**Training of community based providers (LHWs/CMWs/TBAs)**	All **LHWs, and TBAs/CMWs** in the study areas will be trained and sensitized through extensive trainings program to recognize, manage and refer high risk pregnancies and early neonatal problems			Training workshops will be conducted
			**Standard responsibilities**	**Additional responsibilities as part of proposed package**	
		**Antenatal Care**	• Routine antenatal care visits<br>• Detection and management of common pregnancy related problems<br>• Monitoring progress of pregnancy and assessment of maternal & fetal well being<br>• Promotion of institutional deliveries<br>• Education and counseling and referral of complicated cases	• Reinforcement of standard responsibilities<br>• Sprinkle in pregnancy<br>• Equip with mobile sets for effective linkage with community and health facility	▪ Train existing community Midwives and LHWs in the project Areas<br>▪ Conduct routine antenatal care visits<br>▪ Education and Counseling<br>▪ Provision of BP apparatus
		**Natal Care**	▪ Active management of third stage of Labor<br>▪ Referral of complicated cases	▪ Reinforcement of standard responsibilities<br>▪ Assisted skilled Birth through clean delivery kit (if home delivered)<br>▪ Neonatal resuscitation (Ambu bag) to CMWs.<br>▪ Post abortion care<br>▪ Infection prevention	▪ Ensure Skilled Birth attendance<br>▪ Promote institutional delivery<br>▪ Provision of CDK Provision of weighing scale for mother and newborn
		**Post Natal Visit**	▪ Provision of thermal care for low birth weight /pre term babies to prevent hypothermia<br>▪ Monitoring progress of post pregnancy maternal well being<br>▪	• Reinforcement of standard responsibilities<br>▪ Emollient care (sunflower seed oil)<br>▪ Early detection and management of common postnatal problems including First dose administration and facilitated referral<br>▪ Family planning	▪ Conduct 4 Post natal visits (1st 1-2 days, 2nd 5-7 days, 3rd 10-14 days, 4th 40th day)<br>▪ Perform maternal and neonatal examination<br>▪ Education and counseling<br>▪ Provision of antibiotic<br>▪ Provision of weighing scale
For Intervention and control arm	**Health Facility Strengthening**	▪ Strengthening of Public health facilities to encounter pregnancy, childbirth and newborns complications.<br>▪ Development of effective linkage between the field staff, community and health facilities.<br>▪ Training of health care workers in BHU and RHC and THQ,DHQs for basic and comprehensive EmONC<br>▪ Provision of emergency tray at all the health care facilities to prepare and equip them with essential lifesaving drugs (such as adrenalin, hydrocortisone and crystalloids) and equipment (such as laryngoscope and endotracheal tubes)			▪ Linkage will be developed by involving all the relevant stakeholders; Understandings will be built on training, birth attendance and referral mechanisms.<br>▪ Training workshops will be conducted

Supply side interventions

b) ***Enhanced training of LHWs/ CMWs and TBAs:***

The LHWs, CMWs (where present) and TBAs will receive training for early recognition of high risk pregnancies, and identification of need for referral. They will also facilitate transfer to an EmONC facility by identifying and arranging methods of transportation. They would be responsible to stabilize and manage woman/newborn prior to transfer; and communicate with hospital staff for timely and effective management.

c) **Enhanced communication system and referral link:**

LHW and CMWs will be equipped with a communication system (mobile/cell phone) to link her with the community and health facility. Families of pregnant women and newborn babies will be given the number of their area assigned LHW/CMW. In case of emergency, LHW will prepare health staff at the concerned health facility for receiving case she has referred, hence a functional referral link (defined above) will be enhanced between the components of PHC (household/ family, first level health care providers and first level facility), and with EmONC facilities to ensure the required continuity of care throughout pregnancy, childbirth and postnatal care.

d) **Clinical and facility care, strengthening EmONC:**
This will be arranged through training workshops for public sector health care providers of different cadres of intervention and control areas. **Basic Health Units**: Physician/LHV will receive training in basic EmOC and essential newborn care, neonatal resuscitation and management and referral of neonatal problems & complications; **Rural Health Centers**: Physicians and LHV/Nurses will receive training on management of basic and comprehensive EmONC (only where infrastructure will allow and indicate in health facility assessment). **Taluka Hospitals/District Headquarter Hospital**: Physicians (pediatrician and obstetricians)Medical officers, residents LHV/Nurses will receive training on management of comprehensive EmONC with special focus on neonatal resuscitation, management of neonatal sepsis, pneumonia, meningitis, jaundice complications of preterm/LBW and after care of asphyxiated infants.

The curriculum will include training and drills aimed at improving facility responses to emergency conditions such as hemorrhage and eclampsia. Also the health care facilities will be equipped with ambu bag, CDKs, emergency tray and antibiotics (where required).

Presently, health care facilities are either ill equipped or there is lack of knowledge on the utilization and maintenance of supplies. Therefore, we will also institute an **emergency tray** at all the health care facilities existing in the study area to prepare and equip them with essential lifesaving drugs (such as adrenalin, hydrocortisone and crystalloids) and equipment (such as laryngoscope and endotracheal tubes) to deal acute maternal and neonatal emergencies. Also, the staff will be trained to utilize these instruments and knowledge will be provided on its benefits.

Trainings for first level health care providers for community based interventions:

1. Education and IEC material
IEC materials will be developed for the community and families regarding maternal and newborn care, focusing on timely recognition and appropriate responses to newborn danger signs. This material would consist of flip charts. Community and LHW will also be send educational mobile messages to highlight the important aspects of maternal and child health.

2. Training of LHWs of Intervention Clusters
An extensive three-days training workshop will be conducted for LHWs of intervention cluster at initial phase of the study. This training will comprise of guidelines for early recognition of high

risk pregnancies and danger signs in newborns for early domiciliary management and appropriate referral. A major focus would be the motivation of the LHWs to attend births or see the mother and newborn infant soon thereafter. This would require close liaison with the CMWs/TBAs in the area and development of strong linkages between the two cadres of workers. Simple protocols/presentations and job aids will be developed for LHW training in accordance to the current LHW curriculum.

3. Training of TBAs of Intervention Clusters

In context of Pakistan traditional birth attendants (TBAs) have been the main human resource for women during childbirth. Their role varies across cultures and at different times, but even today, they attend the majority of deliveries in rural areas of country. There is little doubt that they have a significant role when it comes to cultural competence, consolation, empathy and psychosocial support at birth with important benefits for the mother and also for the new-born child. In many countries, training TBAs has been an important component of strategies to improve maternal and neo-natal outcomes and same is the case in Pakistan.

An extensive three-day training workshop will be conducted for TBAs of intervention cluster at initial phase of the study. This training will comprise of guidelines for early recognition of high risk pregnancies and danger signs in newborns for early domiciliary management and appropriate referral. TBAs will also be given training on Care of high risk pregnant women, care during delivery newborn care, using the information and referral system and Care during obstetrical or neonatal emergencies in the community.

4. Orientation of CMWs/TBAs of Intervention Clusters

Five days workshops will be organized for CMWs of intervention clusters before launching the field interventions. The training will focus on:

1. Recognition of risk factors for high-risk pregnancies and births (these include maternal malnutrition, illness, short stature, previous perinatal deaths, urinary tract infections and premature rupture of membranes etc.)
2. Clean delivery practices: Importance of using Clean delivery kits
3. Cord care and infection prevention
4. Emollient (sunflower seed oil massage)
5. Immediate newborn resuscitation
    a. They will be trained in neonatal resuscitation using mouth to mouth breathing (for TBAs) and Ambu bag (for CMWs) and strongly encouraged to inform the concerned LHW for linking them up with health facility.
6. Recognition of early post-asphyxia complications for referral.
7. Post abortion care
8. Infection prevention

5. Trainings for facility based Health care providers:

Training packages will be developed for health facility staff. The health care staff of the target health facilities will receive a five day initial training and, where needed, will receive follow-up refresher courses on the screening, recognition, management and referral of antenatal, neonatal

and child health complications. They will also receive training on Emergency Maternal Obstetric Neonatal Care (EmONC) and Essential Neonatal Care (ENC).

Delivery of EmONC Package:

After the training of community and health care providers, the community EmONC package (**discussed in section I**) will be delivered in the intervention clusters primarily through the LHWs and CMWs.

Data Collection Procedures:

Baseline Survey:

a. Household demographic information of the selected area:

A comprehensive baseline survey will be conducted in all 20 Union Councils to collect the baseline characteristics; population socio demography, knowledge, attitude and perceptions of communities regarding maternal and newborn health problems and care. Baseline cross sectional survey will also evaluate births, and maternal and neonatal morbidity and mortality in all intervention and control areas prior to delivery of interventions. The information gathered will also be used for randomization of clusters. The survey will be performed by the separate team of data collectors.

b. Rapid census of health care providers and health care facilities

A rapid census of health care provider and health care facilities will be carried out to pool with the baseline activity to map out the facilities offering maternal, newborn and child health services and to describe the distribution, availability, functioning, and quality of services for MNCH. The study would help us to plan the training activity according to the capacities and service delivery related to obstetric and newborn care. Following information will be gathered through this process:

- General Information
- Staffing
- Activities
- Laboratory & Blood Bank
- Transport & Supplies:
- Problem Prevention/Management Protocols
- Emergency Preparedness in Labor Room & Delivery Suite
- Obstetric Delivery Suite/Postnatal Ward/Neonatal Nursery
- Neonatal Nursery/Intensive Care area

c. Health Facility mapping

Health facility assessment (HFA) is useful for program monitoring and for mapping services to population needs. Following approaches will be used to conduct the assessments.

d. Interviews with Administrator/Manager of Health Facility

Two interviews will be conducted from each health facility in Tehsil RYK. One with the administrator and other with the health care provider, preferably doctor or LHV. The interview with the Health facility administrator/doctor will encompass following domains:

- Existing infrastructure
- Existing health services
- Available human resource
- Modifications required in existing services and resource (human and financial) for improved MNCH outcomes
    - Task shifting/skill mix
    - Expansion of services
    - Integration of service
    - Decentralization of authorities
    - Health care models
    - Budget allocation

e. Client Exit Interviews

Clients will be interviewed as they leave the health facility to measure the effectiveness of the services and to learn about the users' satisfaction of the services of that relevant health facility. Client satisfaction is an important component of good quality of care. Client interviews can reveal how well the facility is performing and the client's perspective on whether or not he/she was treated with respect. Information from client interviews can be used to improve service delivery.

Client exit interviews are not expected to be representative of the population of the whole community since only people who attend the clinic will be interviewed. However, they should be representative of clinic attendees. Therefore the sampling would be systematic and we plan to interview every 5th client coming out of the facility for 3 alternate days.

Routine Record keeping:

The first-level health workers LHWs/CMWs will maintain their own records of visits and maternal and birth outcomes as per standard existing HMIS data collection formats. The health facility staff will keep their record of in and out patient admission with causes and outcome on standardized data collection forms. This exercise will be coupled with the collection of HMIS and MIS data from first level health workers and health facilities by the project staff on monthly basis. The information on referral pattern and tracking of cases will also be documented.

We plan to have a dedicated senior data collection supervisor tasked with collecting and collating data on referrals and allocating household numbers. The main data for analysis will come from the ongoing surveillance for pregnancies, birth outcomes and the household level pregnancy tracking system. As indicated earlier, the two routine systems for data collection from LHW registers and the MIS system from health facilities will be used to complement existing program implementation and feedback by LHW supervisors. We aim to specifically introduce two indicators (LHW attendance at birth and an additional post natal visit within 48 hours) for program monitoring purposes and to provide LHW supervisors with the information for regular follow up with concerned LHWs in the intervention clusters.

This information will also be cross-checked against the data collected independently by the surveillance teams, but will not be the basis for analysis of primary outcomes.

Active Surveillance

We will also conduct active surveillance in the community to obtain vital statistics in the communities of all arms of the study and to evaluate the trends between intervention and control clusters over time. Special data collection teams will be constituted and will work independent of LHWs and other health staff. These data collection teams will collect information from each village on;

- movement of households members and additional population influx
- pregnancies
- live births, stillbirths, neonatal & maternal deaths
- cases of birth asphyxia, sepsis and low birth weight infants,
- Maternal and neonatal referrals etc.

The data will be collected and recorded on standardized forms by a regular system of community visits and also cross-checked by reviewing local hospital records.

At the end we will evaluate the impact of the interventions on the target population. The evaluation will concentrate on impacts of development and implement intervention package for community-level care workers in representative study areas aimed at reducing postpartum maternal and neonatal mortality and morbidity. The results of last surveillance round will be compared with baseline findings, and impact assessment of the interventions will be made on the major domains of evaluation including:

- Design.
- Impact on the primary and secondary outcomes.
- Program efficiency.

Study Outcome & Process Indicators (at the end of each yr (total 3)

The primary outcome variables will be:

- All cause neonatal mortality rate
- Perinatal mortality rate

Secondary outcomes

- All cause maternal mortality
- Cause specific maternal mortality such as deaths related to postpartum hemorrhage, puerperal sepsis, eclampsia, obstructed labor, unsafe abortion and indirect causes
- Maternal morbidity such as (including obstetric fistula, eclampsia and obstetrical sepsis
- Cause specific neonatal mortality such as deaths related to birth asphyxia, prematurity, and sepsis.

The key process indicators include:

1. Proportion of women attended by CMWs/LHWs for ANC

2. Proportion of women supplemented with iron/folate
3. Proportion of women received TT immunization
4. Proportion of births attended by CMW
5. Proportion of births in which LHW was present
6. Proportion of women delivered at institution
7. Proportion of deliveries attended by TBAs
8. Proportion of clean delivery kits used
9. Proportion of women with high-risk pregnancy received referral
10. Proportion of women stabilized or managed before shifting to referral facilities.
11. Proportion of women received comprehensive EmOC services among those who were referred
12. Proportion of newborns seen by CMWs/LHW around day 1, 3, 7 and 28
13. Proportion of newborns resuscitated by CMWs, LHWs and TBAs respectively
14. Proportion of CMWs/LBW able to manage hypothermia
15. Proportion of neonates breastfed in an hour of birth
16. Proportion of neonates avoided pre lacteal feeds
17. Proportion of neonates delayed bathing for at least 6 hours
18. Proportion of neonates who received emollient application
19. Proportion of community participated in support group

Data Management, analysis and reporting:

All data collected will be cross-checked by the field supervisors at field offices on a daily basis before handing over to data management unit (DMU). The data will be transferred to the Field Data Management Center at District RYK from the field stations. Prior to data entry, all forms will be checked for completeness and consistency as well as coding of open- ended responses and area codes, etc. In case of inconsistency or missing responses, the editors will flag the errors/omissions and consult the interviewers for possible explanations. The data entry interface will be designed to check for referential integrity, missing values and acceptability constraints, and there were further checks for heaping at the database level. Errors identified at any level will be referred back to the field for correction.

For data entry, databases and entry screens will be developed using Microsoft FoxPro. The entry screens will employ range and consistency checks and skips to minimize entry of erroneous data. Special arrangements will be made to enforce referential integrity of the database so that all data tables are related to each other without problem. A sub sample of the data will be manually checked to examine data entry errors and to monitor error rates of data entry operators. The data will be double entered.

Analysis of qualitative data

Analysis of qualitative data will give more comprehensive and valid picture for implementation of intervention package. The survey will provide numerical data on distribution of characteristics while the FGDs will provide explanatory insights. Data will be analyzed to develop an understanding of socio-economic, behavioral, situational (i.e., interrelationships, family

dynamics), and environmental (e.g., home, community, etc.) factors that influence a women's choice of having a skilled personnel assisted delivery. Following would be the major themes:

- The barriers and enablers of the skilled care service provision and its uptake in the communities, particularly in poor and marginalized population;
- The role of socio-cultural factors influencing women's choice for birth attendant;
- Quality of care at facilities, community perception on quality of care, support and health workers incentives.

While analyzing data, attention will be given to areas where there are agreements as these may result from censoring of information by participants themselves. This data reduction process will facilitate in identifying the important themes within the data in relation to socio-cultural factors that influence a women's choice to have a skilled delivery. The research team members will have a debriefing session after each focus group session to identify issues which could potential affect analysis (e.g., group dynamics), and identify strategies to improve future focus group sessions.

2. Statistical analysis of Quantitative Data

For data analysis SPSS version 19 will be used and data will be analyzed using univariate and multivariate methods. Reports will be generated fortnightly for senior management of project to review the progress of implementation of interventions.

Primary analysis will be undertaken as intention-to-treat at cluster level. All usual residents of a household who have resided in the study area for >15 days in succession during the 6 months prior to delivery, and delivered during the study period will be considered eligible for analysis, regardless of the place of delivery. Analysis will be done at cluster level using SPSS 19.0 (SPSS Inc., Chicago, Illinois, USA). To account for clustering, point estimates for SBRs, NMRs and perinatal mortality rates for each study arm will be calculated as the mean of cluster event rates, giving an equal weight to each cluster. We will undertake both unadjusted and adjusted analysis of outcomes. We will similarly evaluate differences between intervention and control clusters for cause specific NMR although the study is only powered for the primary all cause neonatal mortality outcomes. Ideally, randomization will take care of confounders, but based on the analysis of baseline characteristics, we will adjust other confounders (if any).

3. Monitoring and Quality Assurance

A standardized system of quality assurance of field procedures and data capture will be instituted. This will be based on daily checks of field procedures, written reports and data entry checks. To ensure proper implementation of the intervention, the study field supervisors will make spot checks and will arrange monthly refresher group sessions of project staff in which the problems encountered will be discussed and resolved. In addition, a 3% sample will be re-interviewed within two days of the initial visit.

Besides quality assurance procedures implemented by the program staff, the monitoring visits will also be conducted by senior project staff, District Coordinator of the LHW program and the

Executive District Health Officer (EDO) District Rahim Yar Khan. During these visits they will record and ensure that:

1. The quality of content, attendance of these trainings;
2. Field activities are going as planned and provide feedback;
3. LHWs are attending deliveries along with TBAs and also managing the newborns immediately after birth and giving them proper referral, if required;
4. The trained health facilities personnel are managing the sick mothers and newborns according to the standard protocols;
5. Availability and stock of the drugs (esp. injection ampicillin and gentamicin) and other supplies through random checks;
6. TBAs and LHWs are well versed in proper interventions or there is any need of refresher;
7. LHWs/TBAs are counseling the family/mother for early breast feeding, maintaining temperature of LBW babies

Q. Ethical Considerations:

Study Design Issues

We have chosen a cluster randomized trial design. This is the most scientifically robust method of assessing comparative public health interventions as compared to other methods such as employing historical controls or using different populations. We do not believe that this design poses any major ethical dilemmas, as the "control" or "intervention" group will continue to receive all the current interventions in place within the primary care program of the government, namely those provided through the Lady Health Workers. In addition, the referral pathway throughout study area will be strengthened. A possible issue is one of contamination and a demand from control areas for interventions. Given the location of these clusters, we will try to minimize this possibility and if it occurs, this will be documented. We are also working to ensure that health facilities are staffed with qualified personnel although the appointment and retention of staff in public health facilities is beyond the control of the project investigators.

Ethical Review Committee (ERC)

The ethical clearance for the entire project would be requested from the Ethical Review Committee at the Aga Khan University. This will be forwarded to Funding agency.

Informed Consent Issues

The investigators will obtain verbal informed consent from all representatives of the communities involved in this study. In addition to these community representatives, we intend to obtain and document verbal community consent from all villages that are participating in this study. The consent will explicitly outline the aims and objectives of the study along with the strict confidentiality of the information provided. In all cases where the newborn is sick, but the parents choose domiciliary care over referral, a written informed consent will be obtained. The informed consent forms describe that participation is voluntary and can be terminated at any time without reason. The scope of the trial is explained. In addition, the parent/guardian will receive information on alternate sources of care same will apply in case of mother is sick and in this case she will give the consent. By agreeing (by signing, when appropriate) to the informed consent form, the parent/guardian confirms the child's or mother confirms her voluntary

participation and their intention to follow the study protocol and the instructions of the investigators.

Environmental consideration

The study anticipate the following Environmental considerations during the flow and implementation of the project:

- The main target audience and beneficiary of this project is the women which usually do not have a say in the decision making and cannot have choices for her to opt for a better treatment. The women usually depend upon the decisions being made by the husbands and elders as the society is male dominant. We have attempted to take care of this factor to maximize the uptake of intervention and services by developing the community support groups.
- The Lack of commitment of the Public Health facility staff and LHWs can largely influence the success of the project. A major factor in sustaining relationships within the local partners is the use of collaborative principles. We will encourage trusting relationships in which there is recognition of the inherent value of all partners' perspectives. We plan to have relationships that foster open communication, development of shared history, coherence of goals, an honest exchange of ideas, and resolution of conflicts which all can contribute to a successful outcome.

R. Research communication and dissemination

At the end of one year of intervention, interim analysis will be undertaken. This interim analysis will focus on relative difference in intervention arms on key study outcomes (morbidity and mortality and process indicators. Findings shall be shared with RAF (along with the formal report) and will be disseminated to stakeholders/ consumers, NGOs, research institutions, government agencies and donor agencies.

The same plan of research communication and dissemination will be repeated on the completion of the study. However, we will make sure to engage and update our stakeholders frequently in following ways

Target Audience: Stakeholders/users (Community groups, NGOs, Research institutions, Government agencies, donor agencies).

We will plan following dissemination activities.

- Research dissemination meetings at district and provincial level involving representatives of community health committees, district level stakeholders, political leaders, civil society organizations
- Sharing of the study reports with MNCH programme and LHW programme both at district and provincial level
- Development of position statements and policy papers at district & provincial level
- Engagement of key stakeholders and actors and build new constituencies of support at district as well as provincial level

- Disseminate and share the evidence with policy makers and stakeholders at provincial level to incorporate in the future provincial policies.

S. Anticipated Utilization or Implications:

It is anticipated that the study will offer a strong message, particularly to policymakers at provincial level, about the need for an integrated approach to develop and implement MNCH

Local level

- We anticipate that awareness regarding maternal and newborn care will increase at individual, family and community levels. This may potentially improve care-seeking for maternal and newborn care.
- We anticipate that this project will increase our understanding of the mechanisms to link the health system and community through community health workers within a functional referral system.

National Level

- Given the burden of maternal and newborn care in Pakistan, the successful outcome of this project will yield information that will be of great value to future training programs for community health workers, and can be replicated in other parts of the country.
- The project will also yield information that will help in determining the balance of community-based and facility-based programs for reducing the burden of postpartum maternal and neonatal complications.
- This project will specifically provide information that will link the maternal and neonatal care components of the existing interventions in this area.
- This project will help in capacity development for partner groups. It's a tailor made process which seeks to strengthen the effectiveness and impact of our program of postpartum maternal and neonatal care.

Global Level

- Maternal and neonatal complication forms a major proportion of maternal and newborn deaths globally. This project will thus provide information that may be of great importance to other health systems and circumstances where a large proportion of births take place in domiciliary settings in the hands of primary care health workers.

T. References:

1. Black RE, Cousens S, Johnson HL, et al. Global, regional, and national causes of child mortality in 2008: a systematic analysis. *The Lancet.* 2010;375(9730):1969-1987.
2. UN. *The Millennium Development Goals Report 2008.* New York: United Nations; 2008.
3. WHO. *The World Health Organisation Annual Report for 2005. Make Every Mother and Child Count.* Geneva: WHO; 2005.
4. Kate J Kerber JEdG-J, Zulfi qar A Bhutta,. Continuum of care for maternal, newborn, and child health: from slogan to service delivery. *Lancet.* 2007.
5. Lawn JE et al. Where is maternal and child health now? Lancet ; . *Lancet.* 2006(368: 1474–77).

6. UNICEF. *THE STATE OF THE WORLD'S CHILDREN 2008.* New York 2008.
7. Bhutta ZA. Rehman S. Perinatal care in Pakistan: a situational analysis. Am J. Perinatology 1997;17:54-9.
8. Bang AT BR, Baitule SB, Reddy HM, Deshmukh MD. Reduced incidence of neonatal morbidities: effect of home-based neonatal care in rural Gadchiroli, India. Management of birth asphyxia in home deliveries in rural Gadchiroli: the effect of two types of birth attendants and of resuscitating with mouth-to-mouth, tube-mask or bag-mask. *Journal of Perinatology.* 2005;25:S82-S91.
9. Jhokio AH, Winter HR, Cheng KK. An intervention involving traditional birth attendants and perinatal and maternal mortality in Pakistan. *New England Journal of Medicine.* 2005;352:2091-2099.
10. Campbell O and Graham W. "Strategies for reducing maternal mortality: getting on with what works" (368): 1284-99. *Lancet.* 2006:(368): 1284-1299.
11. Jose Martines et al. Neonatal survival: a call for action. *Lancet.* 2005.
12. Bhutta ZA, Ali S, Cousens S, et al. Alma-Ata: Rebirth and Revision 6 Interventions to address maternal, newborn, and child survival: what difference can integrated primary health care strategies make? *Lancet.* Sep 13 2008;372(9642):972-989.
13. Adam Wagstaff and Mariam Claeson. *The Millennium Development Goals for Health: Rising to the Challenges.* Washington, DC: World Bank; 2004.
14. Lassi ZS, Haider BA, Bhutta ZA. Community-based intervention packages for reducing maternal and neonatal morbidity and mortality and improving neonatal outcomes. *Cochrane Database of Systematic Reviews.* 2010;Issue 11. Art. No.: CD007754.
15. Syed U, Asiruddin S, Helal SI MI, Murray J. Immediate and early postnatal care for mothers and newborns in rural Bngladesh. *Journal of Heealth, Nutrition and Population.* 2006;24(4):508-518.
16. Darmstadt GL, Choi Y, Arifeen SE, et al. Bangladesh Projahnmo-2 Mirzapur Study Group. Evaluation of a cluster-randomized controlled trial of a package of community-based maternal and newborn interventions in Mirzapur, Bangladesh. *PLoS One.* 2010;5(3):e9696.
17. Bari S, Mannan I, Rahman MA, et al. Bangladesh Projahnmo-II Study Group. Trends in Use of Referral Hospital Services for Care of Sick Newborns in a Community-based Intervention in Tangail District, Bangladesh. *Journal of Health, Population and Nutrition.* 2006;24(4):519-529.
18. Bhutta ZA, Memon ZA, Soofi S, Salat MS, Cousens S, J M. Implementing community-based perinatal care: results from a pilot study in rural Pakistan. *Bulletin of the World Health Organization* 2008;86:452-459.
19. Bhutta ZA, Soofi S, Cousens S, et al. Improvement of perinatal and newborn care in rural Pakistan through community-based strategies: a cluster-randomised effectiveness trial. *Lancet.* 2011;377(9763):403-412.
20. Bhutta ZA, Soofi S, Cousens S, et al. Improvement of perinatal and newborn care in rural Pakistan through community-based strategies: a cluster-randomised effectiveness trial. *Lancet.* 2011;377(9763):403-412.

21. Bhutta ZA, Memon ZA, Soofi S, Salat MS, Cousens S, Martines J. Implementing community-based perinatal care: results from a pilot study in rural Pakistan. *Bulletin of the World Health Organization.* 2008;86:452-459.

22. Midhet F, Becker S. Impact of community-based interventions on maternal and neonatal health indicators: Results from a community randomized trial in rural Balochistan, Pakistan. *Reproductive Health.* 2010;7(1):30.
