# Supplementary material for: Effect of Maternal and Newborn Care Service Package on Perinatal and Newborn Mortality: A Cluster Randomized Clinical Trial
Source: JAMA Netw Open. 2024 Feb 19;7(2):e2356609. doi: 10.1001/jamanetworkopen.2023.56609 (PMC10877450; doi:10.1001/jamanetworkopen.2023.56609)
Supplement: Supplement 2. — eFigure 1. Map of Tehsil RYK eTable 1. Evidence for Each Component of the Intervention Package eTable 2. Enhanced Training for Healthcare Providers eFigure 2. Neonatal Mortality Rate by Arm and Surveillance Round eTable 3. Postpartum Complications eReferences. [file jamanetwopen-e2356609-s002.pdf]

Supplementary Online Content

Ariff S, Jiwani U, Rizvi A, et al. Effect of maternal and newborn care service package on perinatal and newborn mortality: a cluster randomized clinical trial. *JAMA Netw Open.* 2024;7(2):e2356609. doi:10.1001/jamanetworkopen.2023.56609

eFigure 1. Map of Tehsil RYK

eTable 1. Evidence for Each Component of the Intervention Package

eTable 2. Enhanced Training for Healthcare Providers

eFigure 2. Neonatal Mortality Rate by Arm and Surveillance Round

eTable 3. Postpartum Complications

eReferences.

This supplementary material has been provided by the authors to give readers additional information about their work.

© 2024 Ariff S et al. *JAMA Network Open.*

eFigure 1. Map of Tehsil RYK

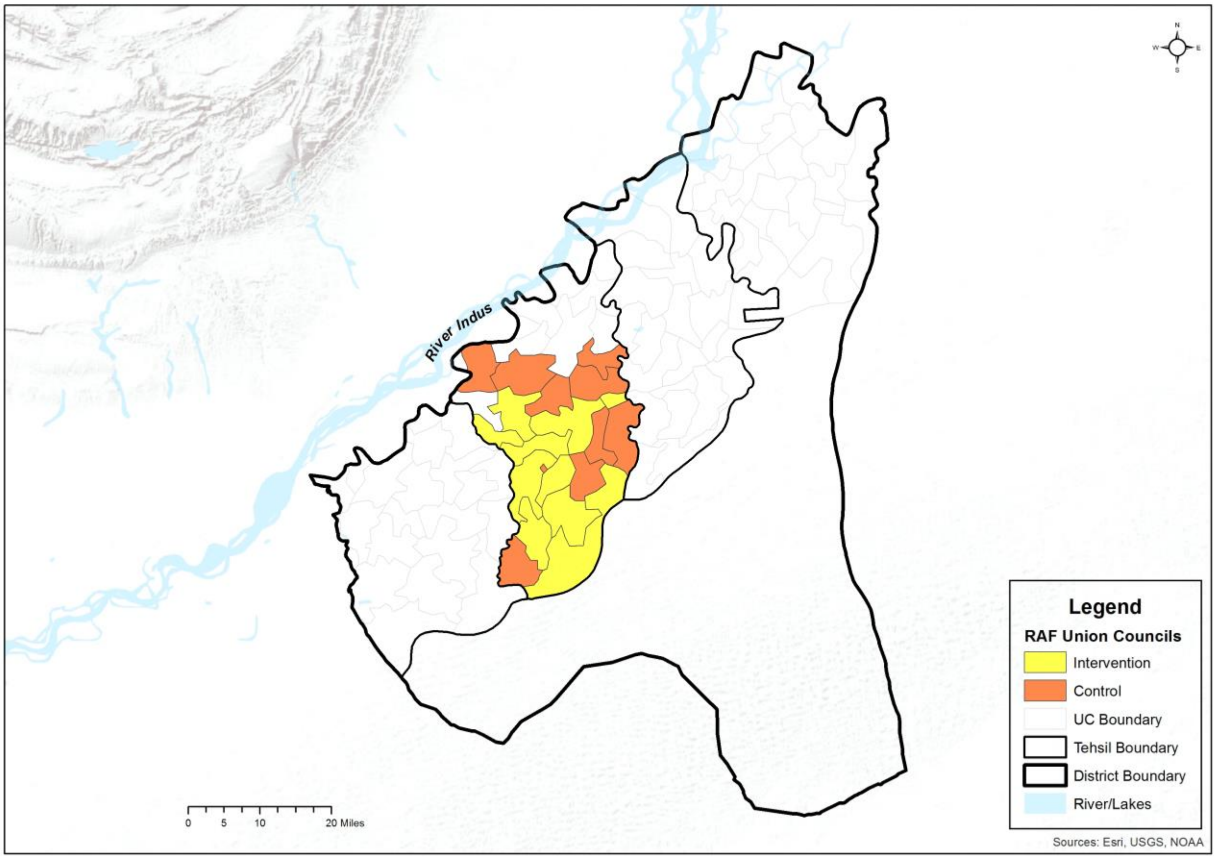

© 2024 Ariff S et al. *JAMA Network Open.*

eTable 1. Evidence for Each Component of the Intervention Package

Intervention component	Evidence
CDK	CDK use can reduce the incidence of omphalitis[1] and increase the use of hygienic practices.[2]
4% Chlorhexidine	A previous cluster randomized trial conducted in district Dadu in Pakistan showed that use of chlorhexidine decreased the risk of neonatal mortality (risk ratio [RR]:0·62, 95 % CI:0·45-0·85) and omphalitis (RR:0·58, 95% CI:0·41-0·82) in neonates.[3] Other trials from Nepal and Bangladesh have similarly reported a reduction in neonatal mortality with the use of chlorhexidine.[4,5]
Sunflower emollient	Sunflower emollients have been shown to reduce the risk of hospital-acquired infections in low-birth-weight neonates.[6,7]
Enhanced training	A multi-country trial showed that training birth attendants in Essential Newborn Care reduced the rate of stillbirths.[8]
Community mobilization	Behavioral change communication interventions including health education, community mobilization, and home-based counseling can reduce the risk of neonatal mortality by 19%.[9]

© 2024 Ariff S et al. *JAMA Network Open.*

eTable 2. Enhanced Training for Healthcare Providers

Cadre of healthcare worker	Description	Standard responsibilities	Enhanced training	Additional responsibilities
Lady health workers (LHWs)	Community health workers employed by the government of Pakistan and receive a monthly stipend. LHWs belong to the same community that they serve and are associated with a healthcare facility within the community. They receive a 15-month-long training on preventing and managing common illnesses in the community. Serve a catchment area of approximately 200 houses.	• Social mobilization, advocacy, and counseling for issues related to better health, hygiene, nutrition, and sanitation. Dissemination of health education message at individual and community levels<br>• Facilitate coordination between health care facilities, NGOs, and community and between local traditional birth attendants and community midwives and other skilled birth attendants for appropriate antenatal, natal, and postnatal services<br>• Registe family members in the catchment area including under-five children and married women aged 15-49 years<br>• Provide family planning services, including counseling, contraceptive provision, and referrals<br>• Provide maternal and child health services including antenatal care services (treatment of anemia, growth monitoring, assessment of common risk factors of malnutrition, nutritional counseling, screening pregnant women to identify those at risk, immunization of pregnant women with tetanus toxoid, helping mothers get access to skilled birth attendants, and proper care during delivery), postnatal services (nutritional education with emphasis on breast-feeding	Received refresher trainings in essential newborn care (ENC) and<br>• Early recognition of high-risk pregnancies, deliveries, and prompt referral<br>• Recognition of danger signs in neonates and prompt referral<br>• Recognition of malnutrition in pregnancy and provision of health education<br>• Early initiation of exclusive breast feeding<br>• Birth weight measurement within 48 hours of birth<br>• Cord care (chlorhexidine application)<br>• Domiciliary management of sepsis and low birth weight (LBW) neonates<br>• Importance and use of clean delivery kits (CDKs)<br>The trial was conducted with full support and cooperation of the LHW program and all LHWs in the intervention districts underwent this training.	• Reinforcement of standard responsibilities<br>• Encourage the use mobile phones (sets) for effective linkage with community and health facility<br>• Ensure skilled birth attendance<br>• Promote institutional delivery<br>• Attend home births and reinforce use of CDKs<br>• Conduct four postnatal care visits on days 1-2, 5-7, 10-14, and 40 to ensure maternal and infant well-being<br>They received adult and newborn weighing scales to record maternal weight during each antenatal care visit and newborn weight at birth. No additional incentives were given.

		and weaning practices, maternal nutrition, and macronutrient malnutrition), and child health (anemia treatment, growth monitoring, immunization)		
Community midwives (CMWs)	Receive an 18-month-long training in midwifery and are then deployed back to their own communities where they establish private practices. Unlike LHWs, CMWs are not present in every village/community. CMWs providing services in the intervention UCs were identified in the baseline surveillance and invited to participate	May vary based on the individual CMW, but in general they<br>• Conduct deliveries using sterile equipment<br>• May facilitate transportation to hospitals and accompany clients to health facilities in emergencies<br>• Provide antenatal care services (administering tetanus toxoid vaccines, counseling women on nutrition and pregnancy complications, assessing, managing, and referring pregnancy complications while also educating pregnant women on symptoms of pregnancy complications)<br>• Provide postnatal care services	Received refreshers on obstetric care using the WHO Essential Antenatal, Perinatal and Postpartum Care training modules with focus on<br>• Identifying high-risk pregnancies<br>• Recognizing danger signs in neonate<br>• Assessing referral indications for mother and neonate and providing prompt referrals<br>• Providing post-abortion care<br>• Clean delivery practices and using CDKs<br>• Cord care and infection prevention	• Encouraged to use the CDKs provided to the pregnant women in the intervention clusters<br>• Encouraged to notify LHW about deliveries
Providers in basic health units (BHUs)	BHUs are primary health facilities. Each UC has a primary health facility that caters to the population of that UC. Providers in BHUs include physicians (medical officers) and nurses (lady health visitors)	BHUs may provide antenatal, delivery (the extent of which depends on the availability of infrastructure such as rooms, medicines such as antibiotics, oxytocin, magnesium sulfate, and equipment, such as forceps and vacuums for assisted vaginal delivery), postpartum, and child care services including immunizations, growth monitoring, management of common infections, and nutritional disorders	Providers in BHUs received refreshers on basic emergency obstetric and neonatal care using the WHO Essential Antenatal, Perinatal and Postpartum Care training modules with focus on<br>• Indications and protocols for administering parenteral antibiotics, uterotonic drugs (oxytocin), and anticonvulsants (magnesium sulfate)<br>• Manual removal of placenta, removal of retained products of conception, assisted vaginal delivery, and neonatal resuscitation<br>• ENC and management and referral of sick newborns	None

© 2024 Ariff S et al. *JAMA Network Open.*

Providers in sub-district hospitals	These are secondary healthcare facilities. The catchment population of these facilities may include more than one UC. Therefore, providers in all 20 UCs were invited to participate in the training	May provide antenatal, delivery (the extent of which depends on the availability of infrastructure such as operating rooms, medicines such as antibiotics, oxytocin, magnesium sulfate, and anesthetics, equipment, such as forceps, vacuums, and epidurals, and human resources, such as anethetists), postpartum, and child care services including immunizations, growth monitoring, management of infections, and nutritional disorders	Providers received refreshers on basic and comprehensive emergency obstetric and neonatal care using the WHO Essential Antenatal, Perinatal and Postpartum Care training modules with focus on<br>• Indications and protocols for administering parenteral antibiotics, uterotonic drugs (oxytocin), and anticonvulsants (magnesium sulfate)<br>• Manual removal of placenta, removal of retained products of conception, assisted vaginal delivery, and neonatal resuscitation<br>• Blood transfusion<br>• Cesarean section<br>• Management of sick and LBW neonates	None

© 2024 Ariff S et al. *JAMA Network Open.*

eFigure 2. Neonatal Mortality Rate by Arm and Surveillance Round

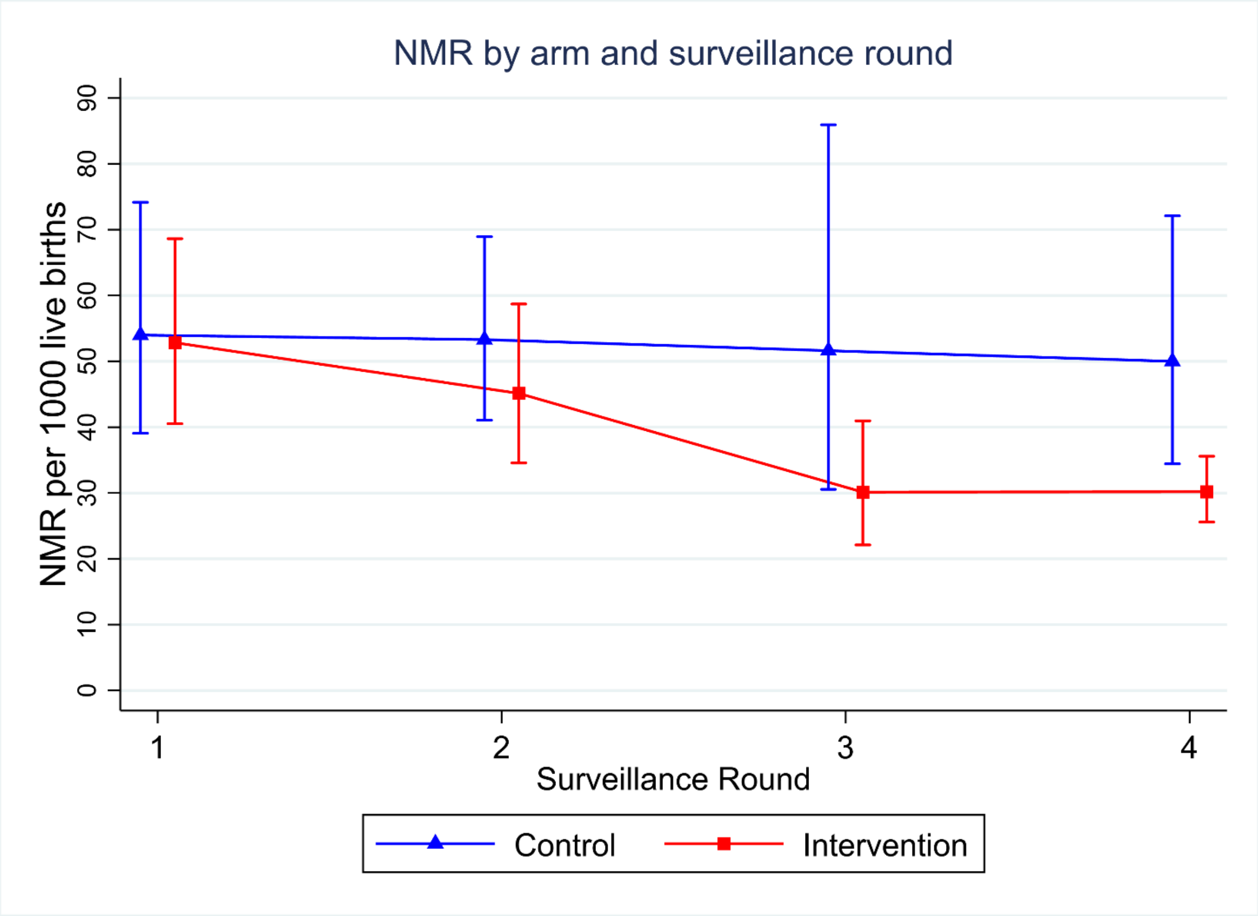

© 2024 Ariff S et al. *JAMA Network Open.*

eTable 3. Postpartum Complications*

	Intervention group (n=7923)		Control group (n=7469)		p-value
	n/N	% (95% CI)	n/N	% (95% CI)	
Any post-partum complications (0-40 days)	815/7923	10.3 (7.0-14.9)	819/7469	11.0 (7.4-15.9)	0.804
Excessive vaginal bleeding	125/815	15.3 (12.5-18.7)	155/819	18.9 (14.6-24.2)	0.176
Ruptured uterus	1/815	0.1 (0.0-1.1)	5/819	0.6 (0.3-1.3)	0.142
Sepsis	9/815	1.1 (0.7-1.8)	9/819	1.1 (0.5-2.3)	0.991
High-grade fever	375/815	46.0 (41.2-50.9)	439/819	53.6 (47.3-59.8)	0.045
Seizures/convulsions/fits	33/815	4.0 (2.8-5.8)	38/819	4.6 (2.7-8.0)	0.665
Unconsciousness	22/815	2.7 (1.8-4.1)	47/819	5.7 (3.7-8.7)	0.009
High blood pressure	36/815	4.4 (2.5-7.6)	20/819	2.4 (1.4-4.1)	0.103
Severe abdominal pain	76/815	9.3 (7.4-11.7)	65/819	7.9 (6.5-9.7)	0.272

*Each woman could have more than one complication

© 2024 Ariff S et al. *JAMA Network Open.*

eReferences.

1. Darmstadt GL, Hassan M, Balsara ZP, Winch PJ, Gipson R, Santosham M. Impact of clean delivery-kit use on newborn umbilical cord and maternal puerperal infections in Egypt. J Health Popul Nutr. 2009;27(6):746-754. doi:10.3329/jhpn.v27i6.4326
2. Balsara ZP, Hussein MH, Winch PJ, Gipson R, Santosham M, Darmstadt GL. Impact of clean delivery kit use on clean delivery practices in Beni Suef Governorate, Egypt. J Perinatol. 2009;29(10):673-679. doi:10.1038/jp.2009.80
3. Soofi S, Cousens S, Imdad A, Bhutto N, Ali N, Bhutta ZA. Topical application of chlorhexidine to neonatal umbilical cords for prevention of omphalitis and neonatal mortality in a rural district of Pakistan: a community-based, cluster-randomised trial. Lancet. 2012;379:1029–1036. doi: 10.1016/S0140-6736(11)61877-1.
4. Mullany LC, Darmstadt GL, Khatry SK, et al. Topical applications of chlorhexidine to the umbilical cord for prevention of omphalitis and neonatal mortality in southern Nepal: a community-based, cluster-randomised trial. Lancet. 2006;367:910–918. doi: 10.1016/S0140-6736(06)68381-5.
5. Arifeen SE, Mullany LC, Shah R, Mannan I, et al. The effect of cord cleansing with chlorhexidine on neonatal mortality in rural Bangladesh: a community-based, cluster-randomised trial. Lancet. 2012;379:1022–1028.
6. Darmstadt GL, Saha SK, Ahmed ASMNU, Chowdhury MAKA, Law PA, Ahmed S, Alam MA, Black RE, Santosham M. Effect of topical treatment with skin barrier-enhancing emollients on nosocomial infections in preterm infants in Bangladesh: a randomised controlled trial. Lancet. 2005;365:1039–1045. doi: 10.1016/S0140-6736(05)71140-5.
7. Darmstadt GL, Badrawi N, Law PA, Ahmed S, Bashir M, Iskander I, Al SD, El KA, Husein MH, Alam A, Winch PJ, Gipson R, Santosham M. Topically applied sunflower seed oil prevents invasive bacterial infections in preterm infants in Egypt. Pediatr Infect Dis J. 2004;23:719–725. doi: 10.1097/01.inf.0000133047.50836.6f.
8. Carlo WA, Goudar SS, Jehan I, et al. Newborn-care training and perinatal mortality in developing countries. N Engl J Med. 2010;362(7):614-623. doi:10.1056/NEJMsa0806033.
9. Tilahun D, Birhanu Z. Effect of community based behavioural change communication intervention to improve neonatal mortality in developing countries: A Systematic Review. JBI Libr Syst Rev. 2011;9(40):1650-1678. doi:10.11124/01938924-201109400-00001

© 2024 Ariff S et al. *JAMA Network Open.*
