# Supplementary material for: Effect of Maternal and Newborn Care Service Package on Perinatal and Newborn Mortality: A Cluster Randomized Clinical Trial
Source: JAMA Netw Open. 2024 Feb 19;7(2):e2356609. doi: 10.1001/jamanetworkopen.2023.56609 (PMC10877450; doi:10.1001/jamanetworkopen.2023.56609)
Supplement: Supplement 3. — Data Sharing Statement [file jamanetwopen-e2356609-s003.pdf]

Data Sharing Statement

Ariff. Effect of Maternal and Newborn Care Service Package on Perinatal and Newborn Mortality. *JAMA Netw Open*. Published February 19, 2024.
doi:10.1001/jamanetworkopen.2023.56609

Data
Data available: Yes
Data types: Deidentified participant data, Data dictionary
How to access data: sajid.soofi@aku.edu
When available: With publication

Supporting Documents
Document types: Statistical/analytic code
How to access documents: sajid.soofi@aku.edu
When available: With publication

Additional Information
Who can access the data: sajid.soofi@aku.edu
Types of analyses: Any purpose
Mechanisms of data availability: A signed data agreement
